# Seed shattering in a North American Oryzeae grain: Developmental and genomic signatures of early domestication

**DOI:** 10.1002/tpg2.70199

**Published:** 2026-02-12

**Authors:** Reneth Millas, Lillian McGilp, Alan Mickelson, Maybell Banting, Claudia Castell‐Miller, Jennifer Kimball

**Affiliations:** ^1^ Department of Agronomy and Plant Genetics University of Minnesota St. Paul Minnesota USA; ^2^ Department of Plant Pathology University of Minnesota St. Paul Minnesota USA

## Abstract

Northern Wild Rice (NWR; *Zizania palustris* L.) is an aquatic grain endemic to North America and a member of the Oryzeae tribe. As an outcrossing crop with a short breeding history, domestication progress in cultivated NWR (cNWR) is ongoing, and seed shattering remains a major barrier to yield stability. In this study, we investigated the developmental and genetic mechanisms underlying seed retention by integrating phenotypic, anatomical, and molecular analyses across wild and cultivated populations. Time‐course phenotyping using four methods revealed a ∼90% reduction and 2‐week delay in shattering in cNWR relative to wild populations. Histological analysis indicated differential anatomical reorganization of the abscission layer in cNWR compared to the wild type. Comparative genomic analyses identified multiple NWR homologs of key *Oryza sativa* shattering genes, revealing lineage‐specific gene duplication, pseudogenization, and divergence from *Zizania latifolia*. Expression profiling of candidate genes via reverse transcription quantitative polymerase chain reaction across floret developmental stages identified the gene *ZpSh5c* as a potential regulator of seed shattering. Together, these findings provide new insights into the anatomical and molecular basis of seed shattering in NWR and demonstrate the utility of comparative frameworks for accelerating trait improvement in emerging, non‐model crops.

## Core Ideas

- Cultivated Northern Wild Rice (cNWR) has a 2‐week delay and approximately 90% reduction in seed shattering at grain maturity compared to the wild type.
- Cellular changes associated with shattering were observed at the seed–pedicle junction of cNWR.
- A whole‐genome duplication in *Zizania* species influences comparative approaches to identify candidate genes of interest.
- The expression pattern of *ZpSh5c* suggests possible involvement in cNWR seed shattering pathways and warrants further investigation.

## Plain Language Summary

Northern Wild Rice (NWR, *Zizania palustris*) has been grown in irrigated paddies in the United States since the 1950s. Seed shattering, that is, when ripe seed separates from the plant pedicel, reduces grain yields in cultivated NWR (cNWR) production. However, little is known about this process. This study compared the seed shattering of two cNWR populations to a wild, unselected population. We found that cNWR seed retention increased ∼90% compared to the time when the wild type shatters, and cNWR began shattering about 2 weeks later than the wild type. Imaging of a layer of cells that lead to shattering at the pedicel, where the seed and stem meet, also revealed differences between cNWR and the wild type. Being closely related to white rice, we leveraged available white rice genomic resources to identify potential NWR genes that may be associated with seed shattering. We found that the gene, *ZpSh5c* may have a possible role in seed shattering in NWR and warrants further research.

**Abbreviations**
ANOVA: analysis of variance
BTS‐B: breaking tensile strength by bending
BTS‐P: breaking tensile strength by pulling
cDNA: complementary DNA
cNWR: cultivated Northern Wild Rice
DAA: days after anthesis
HSD: honestly significant difference
NWR: Northern Wild Rice
PPS: principal phenological stage
RT‐qPCR: reverse transcription quantitative polymerase chain reaction
UMN: University of Minnesota
WGD: whole‐genome duplication

## 1 INTRODUCTION

Seed shattering is a natural seed dispersal mechanism widespread among wild plant species, including the wild ancestors of many modern crops (Konishi et al., 2006; W. Li & Gill, 2006). As species were domesticated, traits that favored seed retention were selected, leading to the replacement of wild‐type alleles with mutations that reduced or eliminated shattering (Konishi et al., 2006; Lin et al., 2012). A key target of this selection was a developmentally regulated abscission event involving the formation and breakdown of an abscission layer, a specialized cell layer, at the seed–pedicel junction (Fuller & Allaby, 2009; Oba et al., 1995; Watanabe et al., 2003). Disruption or loss of this abscission layer decreases seed shattering, significantly improving yields and harvest efficiency, which are critical milestones in crop domestication.

Numerous genes and quantitative trait loci underlying seed shattering have been identified in crop species, particularly in *Oryza sativa* L. and related species (Cai & Morishima, 2000; Ishikawa et al., 2010; Konishi et al., 2006; Lee et al., 2016; Lin et al., 2007; Oba et al., 1995; Thomson et al., 2003; Wu et al., 2017). Several of these have been cloned or fine‐mapped, revealing their roles in regulating the development and function of the abscission layer. Notable examples include the *sh4* gene, which encodes a MYB3 transcription factor (C. Li et al., 2006); *qSH1*, which encodes a BEL1‐type homeobox gene (Konishi et al., 2006); *Sh5*, a highly homologous gene of *qSH1* (Yoon et al., 2014); *SHAT1*, an *APETALA2* (*AP2*) transcription factor (Zhou et al., 2012); and *SH1*, a YABBY transcription factor (Lin et al., 2012). Together, these genes can modulate the development and degradation of the abscission zone and have been repeatedly targeted during the independent domestication of cereal crops.

Across crops, studies have revealed that seed shattering pathways are generally conserved, with many genes either homologous or functionally analogous across divergent lineages (Dong & Wang, 2015; Ishikawa et al., 2022). This pattern of evolutionary convergence reflects similar selection pressures during domestication that were used to increase seed retention (Doebley, 2006; Lenser & Theißen, 2013; Olsen & Wendel, 2013; Purugganan & Fuller, 2009; Tranbarger et al., 2017). Comparative genomic studies have helped to shape our understanding of these convergent evolutionary patterns and are increasingly applied to identify candidate domestication genes in wild and understudied crop species (Fu et al., 2019; Kahler et al., 2014; Liu et al., 2022). For example, recent studies have reported that *SH1* likely plays a role in seed shattering across a wide range of grass species, including *Lolium perenne* (perennial ryegrass), *Setaria italica* (foxtail millet), and *Zizania latifolia* (Manchurian wild rice) (Fu et al., 2019; Liu et al., 2022; Odonkor et al., 2018; Yu et al., 2023). By leveraging such comparative frameworks, researchers can identify conserved regulatory genes and causal variants for key traits, ultimately accelerating the improvement of emerging, non‐model crops.

One emerging crop with strong potential for comparative genomics is Northern Wild Rice (NWR, *Zizania palustris* L.), an annual aquatic grass and crop wild relative of *O. sativa* within the Oryzeae tribe. Endemic to the Great Lakes Region of North America, NWR production in irrigated, diked paddies began in the region in the 1950s. In 1968, the first cultivated NWR (cNWR) variety was released, featuring improved seed retention compared to its wild counterparts (Oelke, 1982). Like *Oryza* species (I. D. Jin et al., 1982, 1990, 1992, 1995; I. D. Jin & Inouye, 1982a, 1982b, 1985), seed shattering in NWR is associated with development of an abscission layer at the seed–pedicel junction composed of parenchyma and sclerenchyma cells, where variation in cell thickness and number contributes to genotype‐specific differences (Hanten et al., 1980). Previous genetic studies have suggested that seed shattering in NWR is a recessive, quantitative trait regulated by at least two or three genes (Elliott & Perlinger, 1977; Kennard et al., 2002; Woods & Clark, 1976). Despite ongoing efforts, modern cNWR varieties and breeding germplasm still exhibit some degree of seed shattering, primarily due to the species’ outcrossing nature and limited breeding history (Elliot, 1980). The trait's persistence contributes to substantial yield losses, particularly after late‐summer storms or high winds (Imle, 2001). As such, NWR offers a valuable system for exploring the genetic basis of seed shattering and applying insights from domesticated relatives to accelerate trait improvement in a semi‐domesticated species.

Improving seed retention in cNWR requires a better understanding of the developmental, anatomical, and genetic factors that regulate seed shattering. Despite its agronomic importance, key aspects of this trait remain underexplored. Given the close phylogenetic relationship between *O. sativa* and NWR, comparative genomics offers a powerful framework for candidate gene discovery (Haas et al., 2021). Therefore, this study aimed to (1) assess the phenotypic variation in seed shattering using four quantitative phenotyping methods; (2) examine the abscission layer anatomy of two cNWR populations and one wild NWR population using histological analysis; (3) identify and compare the protein sequences of putative *O. sativa* homologs in NWR and *Z. latifolia*; and (4) evaluate the expression profiles of putative NWR homologs of *O. sativa* seed shattering‐related genes across stages of panicle development and seed maturation.

## 2 MATERIALS AND METHODS

### 2.1 Plant materials

Three NWR populations were used in this study, including a wild‐type population exhibiting a profuse shattering habit, collected from Sullivan Lake, Minnesota, in 2020 (46.149519 N; −93.936795 W); a cNWR breeding line, FY‐C20, developed for seed length; and a cNWR variety, Itasca‐C12, released in 2007 (Porter et al., 2008), which served as the industry's standard for seed retention at the time of this study. Seeds were removed from cool storage (3°C) (McGilp et al., 2023), rinsed three times, and placed in new plastic bags containing fresh water on a lab benchtop. After 10 days, germinated seedlings were transplanted in the greenhouse.

### 2.2 Plant growth conditions

Experiments were conducted at the University of Minnesota (UMN) Plant Growth Facilities in Saint Paul, MN, in the spring of 2021. Greenhouse conditions were maintained at 22°C (±2°C) with a 16‐h photoperiod. Cone‐tainers (112.98 cm^2^) were filled with a steamed soil mix supplemented with 0.14 g urea (20 lb/acre) and 0.14 g of iron chelate (1 lb/1000 ft^2^), then placed in aluminum aquaponic tanks (66 cm × 183 cm × 70 cm). Tanks were filled with water (13°C) up to 2–4 cm above the surface of the cone‐tainers 1 week before seedlings were planted. To limit algae growth, water was circulated under 5 psi using a 1056 GPH 276 W submersible pump (Simple Deluxe) and treated twice weekly with Algaefix (1 mL/10 gallons of water; API). After germination, one seedling was transplanted per cone‐tainer. Plants were top‐dressed with urea (20 lb/acre) after tillering every 2–3 weeks.

**Core Ideas**

- Cultivated Northern Wild Rice (cNWR) has a 2‐week delay and approximately 90% reduction in seed shattering at grain maturity compared to the wild type.
- Cellular changes associated with shattering were observed at the seed–pedicle junction of cNWR.
- A whole‐genome duplication in *Zizania* species influences comparative approaches to identify candidate genes of interest.
- The expression pattern of *ZpSh5c* suggests possible involvement in cNWR seed shattering pathways and warrants further investigation.

### 2.3 Seed shattering phenotypic data collection

The degree of seed shatter was measured on the main stem panicles of three individual plants per population per timepoint over the course of NWR seed ripening at the principal phenological stage (PPS) 8 (Duquette & Kimball, 2020) using four different methods. Individual plants were collected at their respective days after anthesis (DAA), including 21, 28, 35, and 42 DAA. The first method was a visual estimation of the percentage of shattered seed at the time of data collection, ranging from 0% to 100%, where 0% = *no shatter* and 100% = *complete shatter*. The second method was a drop test, whereby a panicle was dropped three times from a height of 60.96 cm, and the proportion of shattered seeds was calculated relative to the total number of seeds per panicle (Altendorf et al., 2021). The third and fourth methods measured breaking tensile strength using a Chatillon digital force gauge (Ametek, Inc.), either by pulling the seed from the pedicel (breaking tensile strength by pulling [BTS‐P]) or bending (breaking tensile strength by bending [BTS‐B]). For BTS‐P, the seed was gently gripped with forceps attached to the gauge, and force was applied in a straight, downward direction until detachment occurred. The maximum force required to remove the seed was recorded in grams‐force (g), a unit of force where 1 g = 0.0098 N. For BTS‐B, force was applied laterally to the seed at the point of attachment and gently bent until detachment occurred. The force required to break the seed from the pedicel was also recorded in g.

### 2.4 Histology of the seed–pedicel junction

Seed‐pedicel junctions of five plants per population were sampled before reaching the hard dough stage of seed ripening (PPS 85), which corresponded to 21 DAA for the wild‐type population's individuals and 28 DAA for the cultivated line's individuals. Seeds were bisected, placed in a vacuum chamber, and fixed in a mixture of formalin‐acetic acid‐alcohol (53:5:10:32) solution containing 0.1% Triton X‐100. Fixed samples were stored in 70% ethanol at 4°C before being processed at the UMN Histology and Research Laboratory. Upon arrival, the samples were fixed in 2% paraformaldehyde in a 0.1 M phosphate buffer (pH 7.0), dehydrated in an incremental ethanol and ethanol‐xylene series (3:1, 1:1, 1:3, 100% xylene), and embedded in Paraplast Plus (Leica Biosystems). Sections (10 µm) were cut using a rotary microtome, stained with 0.05% toluidine blue O in 0.1 M phosphate buffer (pH 6.8), and imaged using an Axio Scan.Z1 slide scanner (Zeiss Group).

### 2.5 Plant tissue collection

Three plant tissues were collected from three individual plants per population in liquid nitrogen as independent biological replicates. The sampled tissues included flag leaf at the heading stage (PPS 51); male florets at 7 DAA at PPS 66 when all flowering was complete and before pollen shed (PPS 69); and female florets at 1, 7, 18, 28, and 35 DAA throughout seed development (PPS 7) and maturation (PPS 8). In the case of the wild‐type population, female florets were collected only up to 18 DAA, as most seeds had shattered by the next timepoint. Tissue samples were stored at −80°C until further use.

### 2.6 RNA isolation and complementary DNA synthesis

Total RNA was extracted using a RNeasy Plant Mini Kit (Qiagen) following the manufacturer's instructions. RNA concentration and purity were measured using a NanoDrop 2000 spectrophotometer (Thermo Fisher Scientific). To eliminate DNA contamination, RNA samples were treated with the DNA‐free DNA Removal Kit (Thermo Fisher Scientific). Complementary DNA (cDNA) was synthesized from total RNA using the SuperScript III First‐Strand Synthesis SuperMix (Invitrogen, Thermo Fisher Scientific), following the manufacturer's protocol. Synthesized cDNA was stored at −20°C until further use.

### 2.7 Reverse transcription quantitative polymerase chain reaction assays

Gene expression quantification was performed using an Applied Biosystems 7500 Fast Real‐Time PCR System (Thermo Fisher Scientific) on 96‐well plates. Each 20 µL reaction contained 50 ng of cDNA template, 10 µL of iTaq Universal SYBR Green Supermix (Bio‐Rad), and 0.2 µL (10 mM) each of forward and reverse primers (Table S1) using standard thermal cycling conditions (95°C for 20 s, followed by 40 cycles of 95°C for 3 s and 60°C for 30 s). Each gene was evaluated using three biological replicates and two technical replicates per biological replicate, tissue type, and timepoint. Negative controls of no‐template and water as a template were used in all runs.

### 2.8 Selection and evaluation of candidate reference genes for reverse transcription quantitative polymerase chain reaction assays

To identify suitable internal reference genes for reverse transcription quantitative polymerase chain reaction (RT‐qPCR) normalization, five NWR genes were selected as candidates based on their stable expression in other plant species (Joseph et al., 2018; Kozera & Rapacz, 2013) and across eight NWR tissue types (Haas et al., 2021). These included putative homologs of actin (*ACT1* and *ACT4*), eukaryotic translation initiation factor 5 (*EIF5*), glyceraldehyde‐3‐phosphate dehydrogenase (*GAPDH*), and ubiquitin (*UBI*). Primers were designed using PrimerQuest (Integrated DNA Technologies) based on exon sequences extracted from the NWR reference genome v1.0 (Haas et al., 2021) using an in‐house R script. The amplification efficiency and expression stability of each primer set were assessed in leaf, male floret, and female floret tissues across all timepoints. Standard curves were generated using a fivefold serial dilution of cDNA (200, 40, 8, 1.6, and 0.32 ng/µL). Sterile DNase‐ and RNase‐free water was used as a template negative control in all reactions.

### 2.9 Evaluation and selection of putative seed‐shattering homologs

Protein sequences of well‐characterized *O. sativa* seed shattering genes, *SH1*, *qSH1*, *sh4*, *Sh5*, and *SHAT1*, were retrieved from the *O. sativa* ssp. *japonica* reference genome (GenBank accession: GCF_034140825.1). Putative homologs identified in *Z. latifolia* in Yan et al., 2022 were obtained from the Chinese wild rice Jaiobai genome (GCA_0483537475.1; Guo et al., 2015). These *O. sativa* and *Z. latifolia* protein sequences were used as queries in BLASTp searches against the *Z. palustris* Itasca‐C12 reference genome assembly v1.0 (GCA_019279435.1; Haas et al., 2021) using the NCBI BLASTp tool (www.ncbi.nlm.nih.gov/). Candidate homologs in NWR were identified based on percent protein identity with a cut‐off rate of 70%. Orthogroups were inferred using OrthoFinder version 2.5.4 (Emms & Kelly, 2015) and protein domain annotation was performed using Pfam version 37.4 (Finn et al., 2014) via the InterProScan web server (https://www.ebi.ac.uk/interpro/) to identify conserved domains in the predicted protein sequences.

Protein sequences of each gene family were individually aligned using MAFFT v7.511 with the E‐INS‐i algorithm (Katoh & Standley, 2013). The resulting alignments were merged using the –merge function in MAFFT to generate a combined alignment across gene families. A neighbor‐joining tree was generated in MAFFT using default parameters. To visualize conserved residues and domain‐level conservation, protein sequences were aligned using the Constraint‐Based Multiple Alignment Tool (Papadopoulos & Agarwala, 2007) with default parameters. MEME Suite v5.5.5 (Bailey et al., 2009) was used to identify conserved motifs within homologs of each gene. MEME was run in classic discovery mode, restricting the analysis to a single motif (maxsize = 1 or 2) per gene set, focusing on the most highly conserved and potentially functional motifs. Identified motifs were queried against the InterPro database using Pfam version 37.4 to determine correspondence to known Pfam domains. Only motifs with significant hits (*E*‐value < 1e‐5) to known protein domains were considered validated.

### 2.10 Data analysis

To assess variation in seed shattering, an analysis of variance (ANOVA) was conducted in R version 4.3.1 (R Core Team, 2021) for each of the four phenotyping methods across four developmental timepoints. Genotype and timepoint were both considered fixed effects. A statistical significance threshold of 0.05 was used in all analyses. Tukey's honestly significant difference (HSD) tests were performed using the *Agricolae* package version 1.3.7 (de Mendiburu, 2021) to identify pairwise differences among groups. To compare phenotyping methods, Pearson's correlation coefficients were calculated with the *Hmisc* package version 5.2.2 (Harrell & Harrell, 2023).

For reference gene selection, primer efficiency was assessed for each candidate reference gene primer pair by constructing linear regression curves based on the logarithm of the cDNA and the threshold curve cycle (*C*_T_). Primer efficiency (*E*) was calculated from the slope (*S*) of the standard curves generated from serial dilutions, using the equation *E* = [10^(1/−^ *^S^* ^)−^1] × 100% (Rutledge & Côté, 2003). The coefficients of determination (*R* ^2^) were also computed. Expression stability across tissue types and timepoints was evaluated using RefFinder (Xie et al., 2023), which also utilizes geNorm (Hellemans et al., 2008; Vandesompele et al., 2002) and NormFinder (Andersen et al., 2004). The best candidate reference genes with amplification efficiencies within the optimal range (100 ± 10%) and minimal variation in expression were selected for assessing gene expression of candidate shattering genes.

Gene expression levels of seed shattering‐related genes in female and male tissues were quantified using the 2^−∆∆CT^ method (Livak & Schmittgen, 2001). For each sample, threshold cycle (*C*_T_) values of the target gene were first normalized to the selected reference gene to obtain *C*_T_ values. Relative expression was then calculated by comparing normalized *C*_T_ values of each tissue sample to those of the same genes expressed in flag leaf tissue, which was used as the baseline calibrator. The data were transformed using log_2_ and presented as relative gene expression. This approach enabled quantification of fold changes in gene expression relative to a consistent non‐reproductive tissue control. To evaluate differential expression between the wild‐type population and the cNWR populations, ANOVA and Tukey's HSD were also performed.

## 3 RESULTS

### 3.1 Seed shattering variability in NWR populations

To evaluate phenotypic variation in seed shattering, we quantified this trait in three *Z. palustris* populations using four phenotyping methods at 21, 28, 35, and 42 DAA. These methods included visual ratings, a drop test, and two biomechanical tension force methods (BTS‐P and BTS‐B). Statistical analyses (ANOVA, Tukey's HSD; Tables 1 and 2) conducted to assess differences in shattering across populations over their shattering time course revealed that visual ratings and the drop test results were significantly influenced by both genotypes and DAA, while BTS‐P had significant variation by genotype and BTS‐B had no statistical differences (Table 1). In general, visual and drop test scores showed increasing seed shattering over time, while BTS‐B and BTS‐P values, reflecting resistance to detachment, decreased (Table 2). Importantly, differences in the shattering phenotype were not due to variation in developmental timing, as all populations reached comparable phenological stages within 1–3 days of each other (Figure S1).

**TABLE 1:** Analysis of variance (ANOVA) of four phenotyping methods to characterize seed shattering in Northern Wild Rice (NWR; *Zizania palustris*) post‐anthesis.

Method	Source of variation	*df*	Sums of squares	Mean squares	*F*‐value	*p‐*value
Visual	DAA	3	635.42	211.81	5.45	<0.01
	Genotype	2	69,705.56	34,852.78	896.21	<0.0001
	DAA:genotype	6	266.67	44.44	1.14	0.37
	Residuals	24	933.33	38.89		
Drop test	DAA	3	2527.16	842.39	16.01	<0.0001
	Genotype	2	36,198.54	18,099.27	343.97	<0.0001
	DAA:genotype	4	189.28	47.32	0.9	0.48
	Residuals	20	1052.39	52.62		
BTS‐P	DAA	2	18,193.62	9096.81	3.5	0.06
	Genotype	2	33,073.60	16,536.80	6.36	<0.01
	DAA:genotype	2	5465.79	2732.90	1.05	0.38
	Residuals	14	36,419.62	2601.40		
BTS‐B	DAA	2	0	0	0.7	0.51
	Genotype	2	0	0	2.05	0.17
	DAA:genotype	2	0	0	1.1	0.36
	Residuals	14	0	0		

*Note*: A statistical significance threshold of *p* = 0.05 was used.

Abbreviations: BTS‐B, breaking tensile strength by bending; BTS‐P, breaking tensile strength by pulling; DAA: days after anthesis; *df*: degrees of freedom.

**TABLE 2:** Tukey's mean separations analysis for four phenotyping methods to characterize seed shattering in Northern Wild Rice (NWR; *Zizania palustris*) among three populations for each timepoint of collection.

	Visual (%)				Drop test (%)				BTS_pulling (g)				BTS_bending (g)			
**Genotype/DAA**	21	28	35	42	21	28	35	42	21	28	35	42	21	28	35	42
Itasca‐C12	0b	0b	0b	8.33 b	0d	0d	6.25cd	23.23bc	NA	206.78a	168.67ab	142.93ab	NA	0.014a	0.023a	0.01a
FY‐C20	0b	0b	1.67b	13.33 b	0d	0d	10.42cd	35.27b	NA	185.67ab	139.67ab	44.20b	NA	0.027a	0.014 a	0.005a
Wild type	86.67a	98.33a	100a	100 a	91.67a	98.33a	100a	100a	NA	91.33ab	NA	NA	NA	0a	NA	NA

*Note*: Same letters indicate no statistically significant difference (*p* = 0.05). Methods: A visual rating, 0%–100% shattering; a drop test, 0%–100% shattering; breaking tensile strength by pulling (BTS‐P), and breaking tensile strength by bending (BTS_B) were measured in force (g).

Abbreviations: DAA, days after anthesis; NA, not applicable.

For visual ratings, the wild‐type population consistently exhibited greater seed shattering than both cNWR populations, with no significant differences found between Itasca‐C12 and FY‐C20 (Table 2). Shattering in the wild type increased from 86.67% at 21 DAA to 100% by 35 DAA. FY‐C20 began shattering at 35 DAA, reaching 13.3% by 42 DAA, while Itasca‐C12 showed delayed onset, with 8.3% shattering by 42 DAA (Table 2). The drop test also effectively differentiated the wild type from both cultivated populations across all timepoints (Tables 1 and 2). The visual and drop test methods were highly correlated with one another (*r* = 0.99, *p *> 0.001) across all timepoints (Table S3).

For BTS‐P, only one timepoint could be assessed for the wild type, which yielded 91.3 g of force at 28 DAA, compared to 185.7 g for FY‐C20 and 206.8 g for Itasca‐C12 (Table 2). By 42 DAA, Itasca‐C12 required greater force to detach seeds than FY‐C20 (144 vs. 44 g; Table 2). Although BTS‐P differences among populations were not statistically significant, Itasca‐C12 exhibited higher BTS‐P values compared to FY‐C20 across all timepoints. BTS‐P was negatively and significantly correlated with both visual and drop test results across timepoints, except with the visual method at 35 DAA (Table S3). BTS‐B displayed nonsignificant negative correlations with the visual and drop test methods across timepoints. BTS‐B was also nonsignificantly positively correlated with BTS‐P results across timepoints, with one significant correlation between BTS‐B and BTS‐P at 35 DAA (Table S3).

### 3.2 Histological analysis of the seed–pedicel abscission layer

**FIGURE 1:**
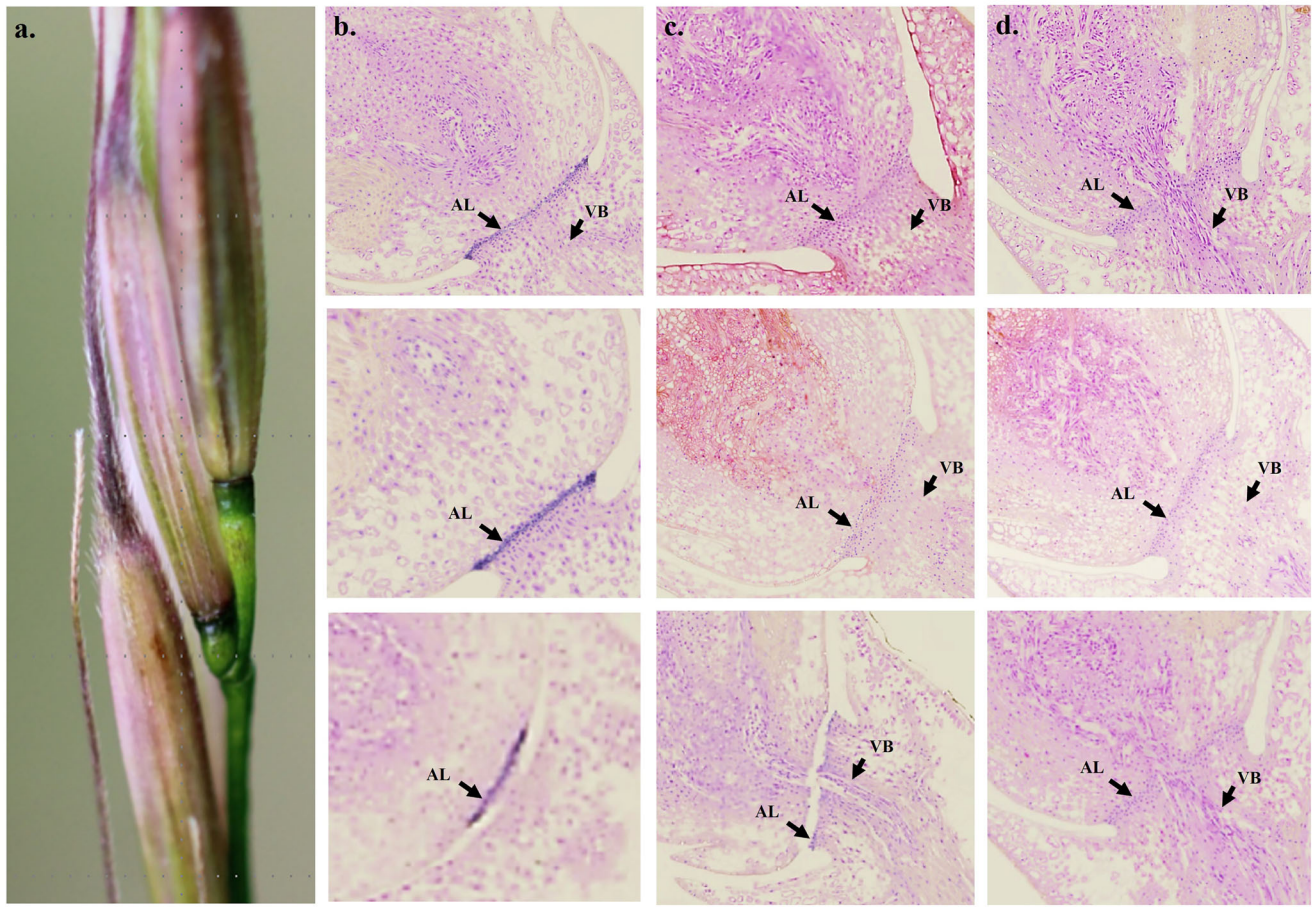
Morphology (a) and abscission layer histology of the seed‐pedicel junctions of three individuals in three Northern Wild Rice (NWR; *Zizania palustris*) populations at the hard dough seed phenological stage, including (b) an unselected wild type representative with a profuse shattering habit, (c) a cultivated NWR (cNWR) population, FY‐C20, with improved seed retention, and (d) the current industry standard cNWR variety for seed retention, Itasca‐C12. AL: abscission layer; VB: vascular bundle. Histological images were observed at a 20X magnification at the plant principal phenological stage 85 (hard dough seed), which corresponded to 21 days after anthesis (DAA) for the wild type and 28 DAA for the cultivated populations.

We examined the anatomical structure of the abscission layer of three individuals in each of the three NWR populations, including a wild‐type population at 21 DAA and two cultivated populations, Itasca‐C12 at 28 DAA and FY‐C20 at 21 DAA. All three populations developed a distinct abscission layer at the seed–pedicel junction (Figure 1). In the wild‐type population (Figure 1b), the abscission layer was well‐defined, consisting of densely packed and structurally organized parenchyma cells among the three individuals. In contrast, the cultivated populations appear to have more loosely arranged cells and larger intercellular spaces disrupting the continuity of the layer. Among the cultivated populations, FY‐C20 showed a more compact and organized abscission layer than Itasca‐C12, which had a more diffused structure and well‐defined vascular bundle (Figure 1c,d, respectively). Also captured was the dehiscence of the wild type and FY‐C20 individuals, as well as the vascular bundles penetrating the seed in the cultivated populations but not the wild type.

### 3.3 Putative relationships and domain structures of seed shattering‐related gene homologs in Oryzeae

Protein sequences of five *O. sativa* genes known to regulate seed abscission layer development, including *SH1* (*OsSH1*), *qSH1* (*OsqSH1*), *Sh5* (*OsSh5*), *sh4* (*Ossh4*), and *SHAT1* (*OsSHAT1*), were queried against the *Z. palustris* genome using BLASTP (Table S4). Multiple homologs were identified for each gene in *Z. palustris*, consistent with a whole‐genome duplication (WGD) in the *Zizania* lineage following its divergence from *Oryza* approximately 26–30 million years ago as well as a rapid expansion of the *Z. palustris* genome within the last 5 million years (Guo et al., 2015; Haas et al., 2021; Yan et al., 2022). Previously reported candidate homologs (Haas et al., 2021) were generally supported by sequence similarity and conserved domain structure. However, for *SHAT1*, *ZpSHAT1a* (ZPchr0004g38673) and *ZpSHAT1b* (ZPchr0458g2256) were stronger candidates than the previously reported ZPchr0013g34051. Additionally, a novel *sh4* homolog (*Zpsh4c*, ZPchr0458g22823) was identified based on high sequence similarity to *ZpSh4a*, *ZpSh4b*, and *Ossh4* (Table 3). Among *O. sativa* and *Z. palustris* proteins, *SH1* had the highest protein identity (PI 89.34%), while *Sh5* had the lowest (71.23%). Between *Z. palustris* and *Z. latifolia*, PI values ranged from 70.00% (*ZpSh5a*) to 98.84% (*ZpSH1*), consistent with the closer evolutionary relationship between the two *Zizania* species.

**TABLE 3:** Percent protein identity (PI) of seed shattering‐related genes in *Oryza sativa* (*Os*) and putative *Zizania palustris* (*Zp*), and *Zizania latifolia* (*Zl*) homologs.

*O. sativa (Os)*		*Z. palustris (Zp)*		*Z. latifolia (Zl)*		Percent identify comparisons between species			Gene description
Gene	GenBank ID	Candidate Gene	GenBank ID	Candidate genes	GenBank IDs (a and b, respectively)	*Zp* vs. *Os*	*Zp* vs. *Zl a/b*	*Os* vs. *Zl a/b*	
*OsSH1*	NP_001389121.1	*ZpSH1*	ZPchr0003g18426	*ZlSH1a/ZlSH1b*	ABZP36_005173/ABZP36_018673	89.84	91.98/98.84	91.94/91.91	YABBY transcription factor (Lin et al., 2012)
*OsSh5*	XP_015637721.1	*ZpSh5a*	ZPchr0001g31104	*ZlSh5a*	ABZP36_013876	71.23	78.85/70.00	89.56/83.92	Bel1‐type homeobox (Yoon et al., 2014)
		*ZpSh5b*	ZPchr0005g15825			84.07	90.05/95.11		
		*ZpSh5c*	ZPchr0010g10516	*ZlSh5b*	ABZP36_030161	87.5	95.64/89.25		
		*ZpSh5d*	ZPchr0010g7757			85.51	95.64/89.25		
*OsqSH1*	XP_015641948.1	*ZpqSH1a*	ZPchr0001g31318	*ZlqSH1a*	ABZP36_025045	83.42	85.44/91.43	85.66	Bel1‐type homeobox (Konishi et al., 2006)
		*ZpqSH1b*	ZPchr0007g5872	*ZlqSH1b*	ABZP36_016879	83.74	90.86/83.28	85.07	
*OsSHAT1*	XP_015636848.1	*ZpSHAT1a*	ZPchr0004g38673	*ZlSHAT1a*	ABZP36_003055	83.65	90.32/88.45	85.74	AP2 transcription factor (Zhou et al., 2012)
		*ZpSHAT1b*	ZPchr0458g22566	*ZlSHAT1b*	ABZP36_007933	84.69	90.30/96.67	84.02	
*Ossh4*	XP_015635337.1	*Zpsh4a*	ZPchr0004g38781	*Zlsh4a/Zlsh4b*	ABZP36_008339s/ABZP36_004261	80	78.05/80.18	76.47/80.12	Trihelix transcription factor (Li et al., 2006)
		*Zpsh4b*	ZPchr0458g22499			79.17	87.41/92.78		
		*Zpsh4c*	ZPchr0458g22823			80.17	86.36/84.21		

*Note*: *Zl a* and *b* genes identified in Yan et al. (2022); *Zl a* and *b* genes GenBank ID in the Jaiobai genome (GCA_0483537475.1; Guo et al., 2015); See Table S4 for protein sequences.

Orthogroup and Pfam analyses provided support for several conserved shattering‐related homologs. *ZpSH1* was identified as a homolog of *OsSH1* and assigned to orthogroup OG0010120, with similar sequences in *Z. latifolia* (Table S5). All homologs contained a YABBY protein domain (Table S6). For *Sh5*, OrthoFinder analysis indicated that *ZpSh5b* was the strongest orthogonal candidate for *OsSh5* within orthogroup OG0013611, which also contained *ZlSh5a* and *ZlSh5b* (Table S5). Two closely related sequences, *ZpSh5c* and *ZpSh5d*, are grouped within orthogroup OGOO26538, with no clear homologs in *O. sativa* or *Z. latifolia*. *ZpSh5a* could not be assigned to an orthogroup. Pfam analysis found that all *Sh5* homologs contain a homeobox domain and a POX domain, except for *ZpSh5a*, which only has a POX domain (Table S6). Comparison of *OsqSH1* identified *ZpqSH1b* as a likely homolog, and it was placed in orthogroup OG0008858, along with *ZlqSH1a* and *ZlqSH1b* (Table S5), while *ZpqSH1a* was not assigned an orthogroup. However, all *Z. palustris* homologs in this group, including *ZpqSH1a*, have homeobox and POX domains (Table S6). For *OsSHAT1*, *ZpSHAT1b* was the strongest orthogonal candidate and grouped in orthogroup OG0003844 with *ZlSHAT1a* and *ZlSHAT1b* (Table S5), while *ZpSHAT1a* was not assigned to an orthogroup. Pfam searches indicated that all *SHAT1* homolog sequences contained two tandem AP2/ERF domains (Table S6). Lastly, *Ossh4*, along with *Zpsh4a*, *Zpsh4b*, *and Zpsh4c* genes, was grouped within the orthogroup OG0004851 (Table S5). All *Z. palustris* homolog sequences for *sh4* contained a Myb/SANT‐like DNA‐binding domain 4 (Table S6).

**FIGURE 2:**
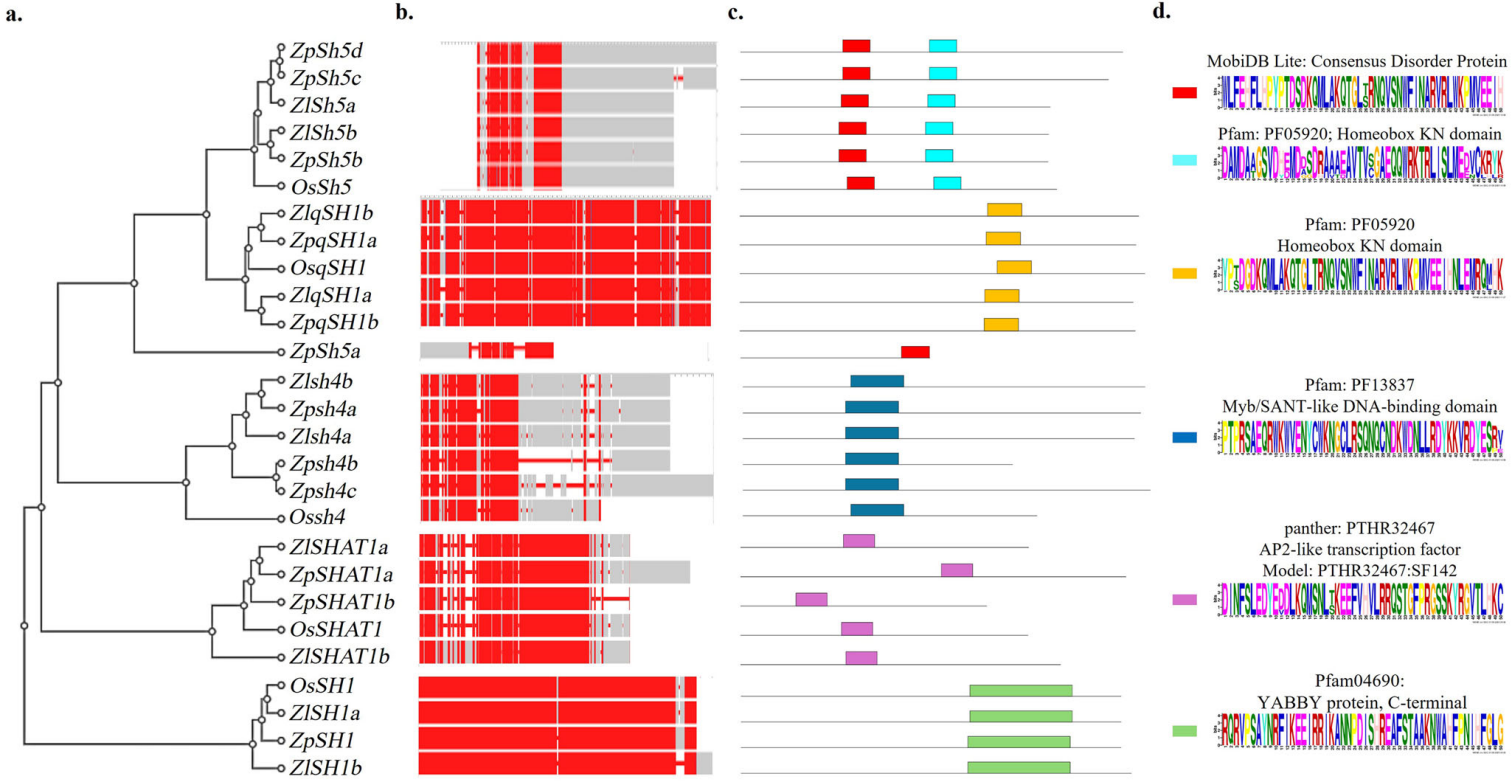
Comparative genomics of seed shattering gene families of *Oryza sativa*, *Zizania palustris*, and *Zizania latifolia*, including (a) a phylogenetic tree, (b) alignments of gene homologs's protein sequences, (c) conserved motifs, and (d) conserved sequences with domain identification. *Os* = *Oryza sativa*, *Zp* = *Zizania palustris*, *Zl* = *Zizania latifolia*; *SH1* = *Shattering1* (Lin et al., 2012), *qSH1* = QTL of seed shattering in chromosome 1 (Konishi et al., 2006), *SHAT1* = *Shattering Abortion1* (Zhou et al., 2012), *sh4* (Li et al., 2006), *Sh5* (Yoon et al., 2014). The tree was built in MAFFT v7.511 with the E‐INS‐i algorithm (Katoh & Standley, 2013) by initially aligning individual gene families followed by assembly into one tree. Protein sequences were aligned using the conserved function in COBALT (Constraint‐based Alignment Tool; Papadopoulos & Agarwala, 2007). Highly conserved areas are highlighted in red. Motifs (= 1 or 2) were identified in MEME Suite v5.5.5 (Bailey et al., 2009) and ran through the Pfam (Finn et al., 2014) database through InterProScan to validate the motif identification.

To continue investigating the genetic relationships and structural conservation of the shattering homologs, a merged neighbor‐joining tree was constructed using individual MAFFT alignments of each gene family, which can be found in Figures S2–S6. This composite phylogeny delineated distinct clades corresponding to each of the five *O. sativa* shattering genes, with *SH1* forming the most distantly related clade (Figure 2a). Gene families such as *Sh5* and *qSH1* grouped closely. Within the *Sh5* cluster, *ZpSh5c* and *ZpSh5d* were most closely related, while *OsSh5* remained within the group but separated from other *Zizania* homologs. Notably, *ZpSh5a* was more distantly placed, separating in the tree prior to the convergence of *Sh5* and *qSH1* clades. The *qSH1* subcluster was characterized by reciprocal groupings of *ZpqSH1a* with *ZlqSH1b* (PI = 91.43%) and *ZpqSH1b* with *ZlqSH1a* (PI = 90.86%), with *OsqSH1* more closely aligned with the former group. The *sh4* subcluster displayed a stepwise branching pattern, where *Zlsh4b* grouped with *Zpsh4a* (PI = 80.18%), followed by *Zlsh4a*, then a branch containing *Zpsh4b* and *Zpsh4c*, and finally *Ossh4*. A similar pattern was observed in the *SHAT1* subcluster, with *ZlSHAT1a* grouping closely with *ZpSHAT1a* (PI = 90.32%), followed by *ZpSHAT1b*, *OsSHAT1*, and finally *ZlSHAT1b*. In the SH1 clade, *OsSH1* grouped most closely with *ZlSH1a* (PI = 91.94%), while *ZpSH1* and *ZlSH1b* diverged into separate terminal branches.

A conserved alignment of protein sequences using COBALT (Constraint‐based Alignment Tool) revealed significant homology across homologs (Figure 2b). Importantly, the alignment revealed that *ZpSh5a* exhibited a truncated protein sequence compared to those of other homologs. MEME analysis detected conserved motifs within gene families, which were validated against the InterPro Pfam database (Figure 2c,d). For example, motifs corresponding to the Homeobox KN domain (Pfam: PF05920) were identified in *qSH1* and *Sh5* homologs, while Myb/SANT‐like DNA‐binding domains (Pfam: PF13837) were identified in SHAT1 homologs. *SH1* homologs displayed conserved YABBY C‐terminal domains (Pfam: PF04690), consistent with previous functional annotations in *O. sativa*.

#### 3.3.1 Reference gene selection for RT‐qPCR experiments

Five commonly used reference genes in plant gene expression studies, *ACT1*, *ACT4*, *EIF‐5*, *UBI*, and *GAPDH*, were evaluated across leaf, female floret, and male floret tissues to identify those with the most stable expression for normalizing RT‐qPCR expression data (Table S1). Primer efficiencies ranged from 106% to 113% with *R* ^2^ values between 0.997 and 0.998 (Table 4). Stability rankings using RefFinder consistently identified *ACT1* as the most stable and *ACT4* as the least stable expressed gene (Table 4). Based on these results, *ACT1* was selected as the internal reference gene for downstream expression analyses.

**TABLE 4:** Primer efficiency and stability of reference gene candidates for gene expression analyses in male and female floret tissues of Northern Wild Rice (NWR; *Zizania palustris*) based on RefFinder, which includes geNorm and NormFinder analyses.

Gene	*Z. palustris* gene	Chr.	Position (bp)	Description	*R* ^2^	%E	RefFinder	geNorm	NormFinder
*ACT1*	ZPchr0012g19383	11	23,232,269	Actin 1	1	110	1.19	0.89	0.94
*ACT4*	ZPchr0010g7718	10	55,029,365	Actin‐related protein 4	1	113	5	4.9	8.9
*EIF‐5*	ZPchr0006g44672	6	52,111,321	Eukaryotic translation initiation factor 5‐like	1	107	1.41	0.89	1.47
GADPH	ZPchr0008g11968	8	9,259,023	Glyceraldehyde‐3‐phosphate dehydrogenase	1	106	3	1.31	1.74
*UBIQ*	ZPchr0007g6160	7	6,416,073	Ubiquitin‐conjugating enzyme E2‐17 kDa isoform X1	1	106	4	2.14	2.13

*Note*: %E, primer efficiency; RefFinder (Xie et al., 2023); geNorm (Hellemans et al., 2008; Vandesompele et al., 2002); NormFinder (Andersen et al., 2004); lower values indicate higher stability and rank.

#### 3.3.2 Expression of *SH1* and *Sh5* homologs in *Z. palustris* female florets

We analyzed the relative expression of NWR candidate homologs of *OsSH1* and *OsSh5*, *ZpSH1* and *ZpSh5a*, *ZpSh5b*, and *ZpSh5c* in NWR female floret tissues (including the seed–pedicel junction) across a time course following anthesis (Table S2). *ZpSH1* was selected due to its high expression in female panicle tissue (Haas et al., 2021) and its conserved role in seed shattering across grass species (Maity et al., 2021). Among four *ZpSh5* putative paralogs, *ZpSh5a*, *ZpSh5b*, and *ZpSh5c* were included based on their relatively high expression levels (Haas et al., 2021) and to explore their potential roles in the regulatory pathways of seed shattering, considering their putative diversification following the WGD. *ZpSh5d* was omitted from RT‐qPCR assays due to its low expression in eight NWR tissues (Haas et al., 2021).

**FIGURE 3:**
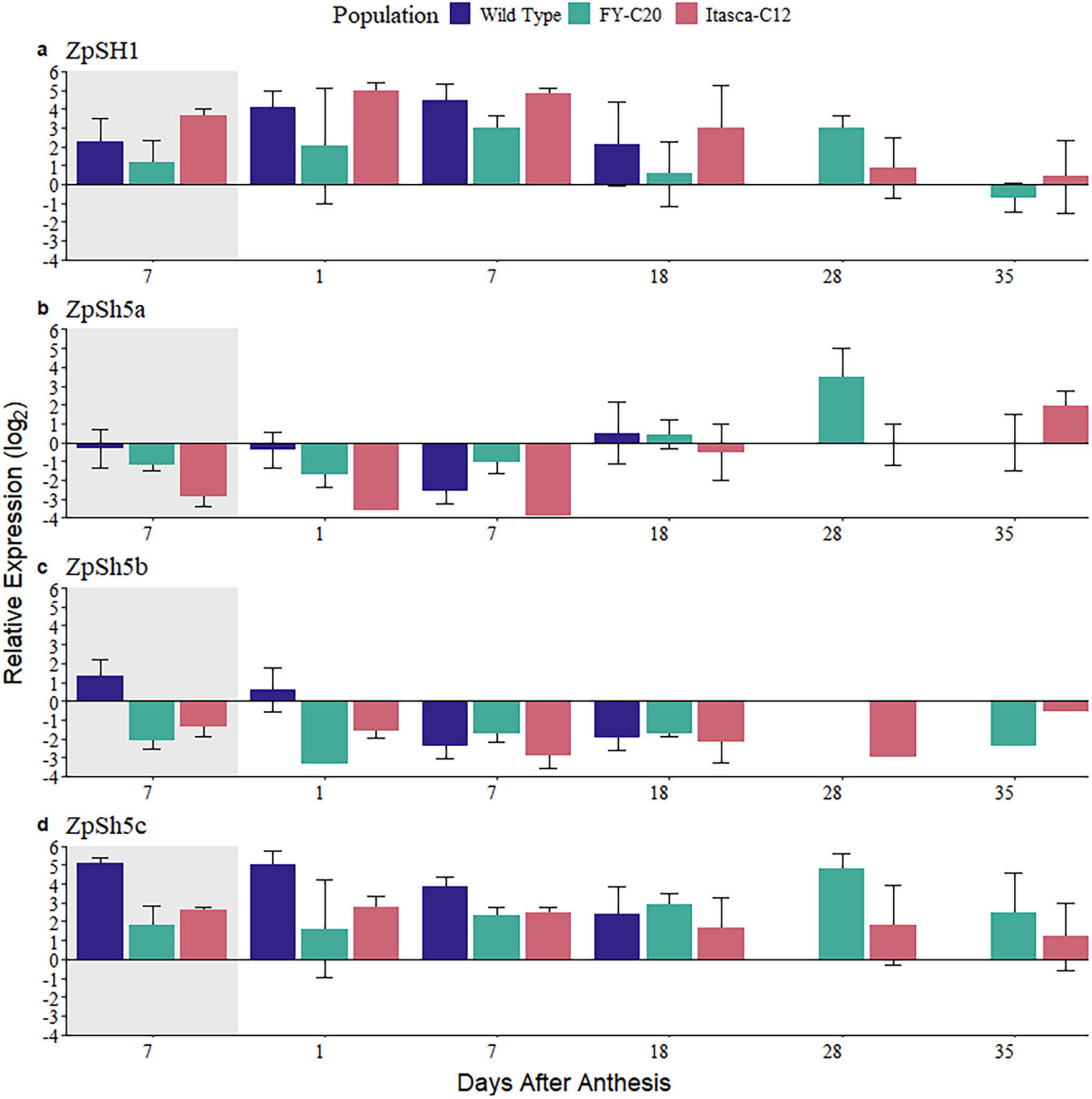
Relative expression of candidate seed‐shattering genes in female and male floret tissues of Northern Wild Rice (NWR; *Zizania palustris*) post‐anthesis across a wild type and two cultivated populations. *Zp* = *Zizania palustris*, *qSH1* = QTL of seed shattering in chromosome 1 (Konishi et al., 2006), *Sh5 *= (Yoon et al, 2014). Log2 relative gene expression was measured in male florets collected at 7 days after anthesis (DAA) (indicated in gray) and in female florets collected at 1, 7, 18, 28, and 35 DAA across four genes.

To characterize candidate gene expression, female florets were collected at 1, 7, 18, 28, and 35 DAA for RT‐qPCR analysis. Data from the wild‐type population were unavailable at 28 and 35 DAA due to near‐complete seed shattering (98%) by 28 DAA (Table 2). The RT‐qPCR gene expression time course profiles of female florets are summarized across genes in Figure 3 and by population in Figure S2. *ZpSH1* was upregulated compared to leaf tissue, except for FY‐C20 at 35 DAA (Figure 3a). Across DAA, the expression of *ZpSH1* in the wild type was high on 1 DAA, peaked at 7 DAA, and declined at the last timepoint of the population's collection, 18 DAA. FY‐C20's expression was lower than the wild type from 1 to 18 DAA but with peaks at 7 and at 28 DAA and was slightly downregulated by 35 DAA. Itasca‐C12 had higher expression than the wild type from 1 to 18 DAA, peaking at 1 DAA and then declining through the rest of the timepoints. For *ZpSh5a*, expression in female floret tissues was downregulated at 1 and 7 DAA for all populations compared to leaf tissue (Figure 3b). Expression of the wild type was slightly upregulated at 18 DAA, its final timepoint. The expression of *ZpSh5a* in FY‐C20 after 7 DAA was upregulated with a peak at 28 DAA and undetectable at 35 DAA. In Itasca‐C12, the gene was downregulated from 1 DAA through 18 DAA, undetectable at 28 DAA, and upregulated at 35 DAA. The expression of *ZpSh5b* in female floret tissue was downregulated compared with leaf tissue for all populations and timepoints except for the wild type at 1 DAA (Figure 3c). The expression of *ZpSh5c* in female floret tissue was upregulated for all populations and timepoints compared with leaf tissue (Figure 3d). The expression in the wild type was highest at 1 DAA, then declined through 18 DAA. Itasca‐C12 followed a similar pattern of expression as the wild type, though with lower expression, peaking at 1 DAA, with lower expression through the rest of the timepoints. However, FY‐C20 peaked at 28 DAA, about 3 weeks later than the wild type (Figure 3d).

ANOVA conducted on a per‐gene basis revealed that genotype was not significant for any of the genes (Table 5). Expression of *ZpSH1* and *ZpSh5a* was influenced by DAA; *ZpSH1* expression at 7 DAA was different from 35 DAA, and *ZpSh5c* expression at 1 and 7 DAA was different from 28 DAA (Table S7). No significant differences between populations or timepoints were observed for candidate genes *ZpSh5b* and *ZpSh5c*. ANOVA conducted on a per population basis revealed significant differences between gene expression for all populations, where *ZpSH1* and *ZpSh5c* had higher expression than *ZpSh5a* and *ZpSh5b* (Table 5; Table S7). For FY‐C20, there were also significant differences between timepoints; 28 DAA was significantly different from 1 DAA and 35 DAA. For Itasca‐C12, the interaction between gene and DAA was significant. Overall, *ZpSH1* had higher expression, with 1 and 7 DAA being significantly higher than *ZpSh5b* at 7, 18, and 28 DAA and also *ZpSh5a* at 1 and 7 DAA. The expression for all timepoints of *ZpSh5c* was intermediate and did not differ significantly from any other genes (Table S7).

**TABLE 5:** Analysis of variance (ANOVA) analyses of the relative gene expression of four Northern Wild Rice (NWR; *Zizania palustris*) candidate homologs of *Oryza sativa* genes related to seed shattering in three NWR populations.

Gene	Source of variation	*df*	Sum of squares	Mean squares	*F*‐value	*p*‐value
*ZpSH1*	DAA	4	83.44	20.86	2.87	0.044
	Genotype	2	12.93	6.46	0.89	0.424
	Replication	1	10.69	10.69	1.47	0.237
	DAA:genotype	6	23.95	3.99	0.55	0.766
	Residuals	25	181.9	7.28		
*ZpSh5a*	DAA	4	94	23.5	6.66	0.001
	Genotype	2	23.35	11.68	3.31	0.054
	Replication	1	4.13	4.13	1.17	0.29
	DAA:genotype	6	28.58	4.76	1.35	0.275
	Residuals	24	84.73	3.53		
*ZpSh5b*	DAA	4	6.26	1.56	0.44	0.777
	Genotype	2	6.58	3.29	0.93	0.415
	Replication	1	1.02	1.02	0.29	0.6
	DAA:genotype	5	20.97	4.19	1.18	0.36
	Residuals	16	56.69	3.54		
*ZpSh5c*	DAA	4	8.35	2.09	0.4	0.806
	Genotype	2	16.89	8.44	1.62	0.221
	Replication	1	20.25	20.25	3.89	0.062
	DAA:genotype	6	22.38	3.73	0.72	0.641
	Residuals	21	109.36	5.21		

Genotype	Source of variation	*df*	Sum of squares	Mean squares	*F*‐value	*p*‐value
Wild type	DAA	2	18.91	9.46	2.35	0.118
	Genes	3	201.23	67.08	16.66	<0.001
	Rep	1	0.09	0.09	0.02	0.882
	DAA:genes	6	32	5.33	1.32	0.286
	Residuals	23	92.61	4.03		
FY‐C20	DAA	4	85.9	21.48	3.51	0.018
	Genes	3	121.08	40.36	6.59	0.001
	Rep	1	19.06	19.06	3.11	0.088
	DAA:genes	11	36.53	3.32	0.54	0.859
	Residuals	31	189.89	6.13		
Itasca‐C12	DAA	4	4.99	1.25	0.27	0.892
	Genes	3	245.14	81.71	18	<0.001
	Rep	1	26.69	26.69	5.88	0.021
	DAA:genes	12	117.75	9.81	2.16	0.04
	Residuals	33	149.82	4.54		

Abbreviations: DAA, days after anthesis; *df*, degrees of freedom.

#### 3.3.3 Expression of *SH1* and *Sh5* homologs in male florets

To investigate potential overlap in the regulatory mechanisms between male and female floret shattering, we analyzed expression in male florets at 7 DAA. Similar to the expression patterns in female florets, *ZpSh1* and *ZpSh5c* were upregulated in all populations (Figure 3). Expression was highest in Itasca‐C12 for *ZpSH1* and in the wild type for *ZpSh5c*. At 7 DAA, the expression of *ZpSh5a* in male florets was downregulated for all populations, consistent with the early DAA in female florets. For *ZpSh5b*, wild‐type male florets were upregulated, while the cultivated populations were downregulated, consistent with the expression patterns of female florets at 1 DAA (Figure 3). When averaged across genes and populations, the male floret expression at 7 DAA was significantly correlated with 1 (0.82), 7 (0.80), and 18 (0.38) DAA in the female floret tissue (Table S8).

## 4 DISCUSSION

### 4.1 Selection‐driven changes in cNWR seed retention

Seed shattering is a key adaptive trait in wild grasses, enabling seed dispersal, but it presents a major obstacle in grain domestication. In cNWR, strong selection pressure for seed retention has modified the timing and structure of seed abscission, despite a short domestication history. Our results show that cultivated populations exhibited a 90% reduction and 2‐week delay in shattering relative to the wild type (Table 2), possibly due to alteration of the abscission zone. Histological observations support this, where cultivated populations displayed less organized and compact abscission layers, consistent with delayed or disrupted cell separation processes (Figure 1). The changes observed in this study were also parallel to those seen in domesticated *Oryza* species (Ji et al., 2006; I. Jin & Inouye, 1981; X. Li et al., 2024; Lin et al., 2007; Oba et al., 1995; Thurber et al., 2013) and suggest that similar cellular mechanisms are being targeted by selection in NWR. The observed changes in tensile strength and shattering progression, particularly in the higher seed‐retaining Itasca‐C12 (Table 2), suggest population‐specific modifications to abscission layer timing or robustness, reflecting the ongoing selection for this trait. While this study identified a potential relationship between seed shattering and the development of cNWR's abscission layer, further investigation into the timing of abscission layer development and degradation is needed, as histological assessments were limited to a single timepoint per population and did not include the connectivity of the vascular bundle from pedicel to the grain for all populations.

### 4.2 Gene duplication and divergence underlie shattering variation

Gene duplication and divergence are central to trait evolution following WGD events. Our comparative genomic analyses revealed that NWR harbors multiple homologs, similar to *Z. latifolia* (Yan et al., 2022), of known *O. sativa* seed shattering genes, including *sh4*, *SHAT1*, *qSH1*, and *Sh5*. This redundancy, arising from a WGD and subsequent gene expansion in NWR (Haas et al., 2021), suggests that regulatory divergence rather than gene presence/absence may drive variation in NWR seed shattering. For example, *SHAT1* homologs showed evidence of divergence in NWR. *ZpSHAT1b* clustered with known functional homologs, while *ZpSHAT1a* did not cluster with any known orthogroup, possibly reflecting neofunctionalization or regulatory repurposing (Wang et al., 2012). The exception was a single functional homolog of *OsSH1*, *ZpSh1*, which retained a YABBY transcription factor and consistently clustered with homologs from *O. sativa* and *Z. latifolia* (Table S5), indicating its conserved role in abscission regulation.

In particular, *ZpSh5*, a Bel1‐homeobox domain, exhibited paralog divergence among four strong *Sh5* candidates. *ZpSh5a*’s loss of a key homeobox domain, for example, suggests it may be transitioning toward pseudogenization (Panchy et al., 2016). The retention of multiple *Sh5‐*like genes, with varying degrees of structural and functional integrity, reflects the history of WGD and subsequent segmental or transposon‐mediated duplications (Fedoroff, 2012; Huang et al., 2022; Leister, 2004; Wang et al., 2012). The differential expression of *ZpSh5* paralogs also supports a role for subfunctionalization or regulatory partitioning in NWR genes following duplication. While some copy number differences between *Z. palustris* and *Z. latifolia* could be due to assembly artifacts, others likely reflect true genomic expansion, consistent with the larger genome size of *Z. palustris*. This complexity highlights the challenge of linking genotype to phenotype in recently duplicated genomes and the need for functional assays to dissect roles of individual paralogs.

### 4.3 Developmental timing of gene expression differentiates shattering phenotypes

Developmental regulation of gene expression is a key determinant of phenotypic variation in domestication traits such as seed shattering. In this study, we identified a general trend of higher expression of *ZpSH1* and *ZpSh5c* relative to the other tested genes. *ZpSH1* exhibited higher expression in the early DAA compared to later DAA (Figure 3a), consistent with *SH1*’s known role in promoting abscission layer formation in grasses (Lin et al., 2012; Yu et al., 2023) and supports the hypothesis that *ZpSH1* functions as a conserved regulator of shattering in NWR. *ZpSh5c* also exhibited higher expression in the early DAA in the wild type and in Itasca‐C12, while the gene's expression peaked later, at 28 DAA, in FY‐C20 (Figure 3d). This peak expression occurred 7 days before the onset of shattering in FY‐C20, which may be consistent with *Sh5*’s known role in regulating abscission zone differentiation and downstream enzymes (Yoon et al., 2014). These findings support a model in which selection for seed retention in cNWR has acted on regulatory changes, such as the timing and magnitude of gene expression (e.g., abscission layer morphology), and that population‐specific responses to artificial selection vary in cNWR.

While sample size was limited, strong correlations between male and female floret expression in the early DAA also suggest that these tissues may have, at least initially, similar shattering mechanisms. In NWR, male floret shattering occurs prior to that of the female florets and has been used as an early, indirect selection tool for improving seed retention (Kennard et al., 2002). These results reinforce the utility of this selection method in breeding programs.

Overall, the developmental and regulatory changes identified in this study resemble patterns observed in *Oryza*, where domestication involved sequential, quantitative shifts in expression across shattering‐related loci (Ishikawa et al., 2022). We cannot rule out unidentified interactions between these and other unexamined genes, which may modulate seed shattering in NWR and warrant continued research to fully elucidate mechanisms of seed shattering in this native North American species.

## 5 CONCLUSIONS

Despite the short breeding history of cNWR, signatures of the reduction of seed shattering were observed in this study at the anatomical, physiological, and gene expression levels. Changes in abscission layer development, increased tensile strength, and altered expression of key regulatory genes collectively support the hypothesis that selection has reshaped the developmental and genetic control of shattering in cNWR. Comparative genomic analyses revealed an increased number of homologs of *O. sativa* shattering genes (e.g., *OsSh5*), consistent with the WGD history and genomic complexity of *Z. palustris*. Among these, *ZpSh5c* showed biologically relevant expression variation across populations, suggesting roles in modulating abscission zone formation and seed retention. *ZpSh5c*’s delayed expression in one of the cultivated populations aligns with its delayed shattering phenotype and may reflect a target of selection. Together, these findings highlight the potential of cNWR as a model for investigating the early stages of domestication. Its phylogenetic proximity to *Oryza* and ongoing directed selection offer a rare opportunity to study the molecular evolution of domestication traits in real time. Further integrative studies across broader germplasm, incorporating functional assays and regulatory network analysis, will be key to understanding the complex genetic basis of seed shattering in this North American cereal.

## AUTHOR CONTRIBUTIONS

**Reneth Millas**: Data curation; formal analysis; investigation; methodology; visualization; writing—original draft. **Lillian McGilp**: Data curation; software; validation; visualization; writing—review and editing. **Alan Mickelson**: Conceptualization; data curation; methodology; project administration; resources; validation; writing—original draft; writing—review and editing. **Maybell Banting**: Data curation; formal analysis; investigation; validation; writing—review and editing. **Claudia Castell‐Miller**: Conceptualization; investigation; writing—review and editing. **Jennifer Kimball**: Conceptualization; funding acquisition; project administration; supervision; visualization; writing—original draft; writing—review and editing.

## CONFLICT OF INTEREST STATEMENT

The authors declare no conflicts of interest.

## Supporting information



Supplementary Tables and Figures

## Data Availability

Most data are provided within the manuscript or Supporting Information files. Other raw data files associated with this project have also been submitted to the Data Repository for the University of Minnesota (DRUM) and can be accessed via doi: https://doi.org/10.13020/q7w7‐gj62.
