# Supplementary material for: Seed shattering in a North American Oryzeae grain: Developmental and genomic signatures of early domestication
Source: Plant Genome. 2026 Feb 12;19(1):e70199. doi: 10.1002/tpg2.70199 (PMC12903962; doi:10.1002/tpg2.70199)
Supplement: Supplementary file 1 [file TPG2-19-e70199-s001.docx]

**SUPPLEMENTARY TABLES AND FIGURES**

**Supplementary Table 1.** Primer sequences for candidate internal reference genes in Northern Wild Rice (NWR; *Zizania palustris*).

| ***O. sativa* GenBank ID** | ***Z. palustris* GenBank ID** | **Chr** | **Gene description** | **Forward primer (5' to 3')** | **Reverse primer (5' to 3')** |
| --- | --- | --- | --- | --- | --- |
| NP_001409835.1 | ZPchr0010g7718 | 10 | Actin-related protein 4 | GAGGTCTCGGCCATCGT | AGGAGAAGACGGCCTTGG |
| XP_015643055.1 | ZPchr0006g44672 | 6 | Eukaryotic translation initiation factor 5-like | GAAACAGGAGGTCCCACATAAG | TCTCTTCAACCAGGTCATCATAAA |
| NP_001390311.1 | ZPchr0008g11968 | 8 | Glyceraldehyde-3-phosphate dehydrogenase | CCTTCATCACCACCGAATACA | GGTGTGCACGCAAACATATC |
| XP_015621235.1 | ZPchr0007g6160 | 7 | Ubiquitin-conjugating enzyme E2-17 kDa isoform X1 | GAGAGGCACGCCAACTTTA | AGATCTAGGAGCCGGAGTTC |
| NP_001389172.1 | ZPchr0012g19383 | 11 | *O. sativa Actin 1* | CTGTGGAGAAGAGCTATGAGTTG | CTACGAAAGATGGCTGGAAGAG |
| *Annotations and chromosome numbers are based on the Z. palustris assembly v. 1.0 (Haas et al., 2021) | | | | | |

**Supplementary Table 2.** Primer sequences for candidate *Zizania palustris* homologs of *Oryza sativa* genes used in RT-qPCR assays.

| **Gene Name** | **Gene ID** | **Zp Genbank ID** | **Description** | **Forward Primer** | **Reverse Primer** |
| --- | --- | --- | --- | --- | --- |
| *ZpSH1* | ZPchr0003g18426 | KAG8062892 | YABBY transcription factor-like | ATGTCAGCACAGATCGCA | CGCGAGAATCGTGTTGC |
| *ZpSh5a* | ZPchr0001g31104 | KAG8052160 | Bel1-homeobox domain protein 9 | ATGAGGGAGATGAGCCTAGTT | TCGACCACGAGATGGATGA |
| *ZpSh5b* | ZPchr0005g15825 | KAG8068426 | Bel1-homeobox domain protein 9 | ATGAGCCTAGTCTTCCTCCA | CGAGGGTTTGCTCGACTTAG |
| *ZpSh5c* | ZPchr0010g10516 | KAG8085781 | Bel1-homeobox domain protein 9 | GGATGTCAGTGATCCCTATGTG | CCTACAAAGTCATGGAGCAAATG |

**Supplementary Table 3.** Pearson correlation coefficients and corresponding p-values for four methods evaluating seed shattering resistance in Northern Wild Rice (NWR; *Zizania palustris*) at different days after anthesis (DAA). Methods included visual ratings, a drop test, and two breaking tensile strength tests by pulling (BTS-P) and bending (BTS-B).

| DAA |  | 28 | | | | 35 | | | | 42 | | | |
| --- | --- | --- | --- | --- | --- | --- | --- | --- | --- | --- | --- | --- | --- |
|  | Methods | Visual | Drop | BTS_P | BTS_B | Visual | Drop | BTS_P | BTS_B | Visual | Drop | BTS_P | BTS_B |
| 28 | Visual |  |  |  |  |  |  |  |  |  |  |  |  |
|  | Drop | 1.00*** |  |  |  |  |  |  |  |  |  |  |  |
|  | BTS_P | -0.72* | -0.72* |  |  |  |  |  |  |  |  |  |  |
|  | BTS_B | -0.65 | -0.65 | 0.40 |  |  |  |  |  |  |  |  |  |
| 35 | Visual | 1.00*** | 1.00*** | -0.70* | -0.65 |  |  |  |  |  |  |  |  |
|  | Drop | 0.99*** | 0.99*** | -0.69* | -0.72* | 0.99*** |  |  |  |  |  |  |  |
|  | BTS_P | -0.89** | -0.89** | 0.63 | 0.46 | -0.90** | -0.84** |  |  |  |  |  |  |
|  | BTS_B | -0.50 | -0.50 | 0.36 | -0.06 | -0.49 | -0.41 | 0.76* |  |  |  |  |  |
| 42 | Visual | 0.98*** | 0.98*** | -0.68* | -0.68* | 0.97*** | 0.99*** | -0.84** | -0.48 |  |  |  |  |
|  | Drop | 0.96*** | 0.96*** | -0.68* | -0.53 | 0.96*** | 0.95*** | -0.87** | -0.62 | 0.98*** |  |  |  |
|  | BTS_P | -0.70* | -0.70* | 0.58 | 0.26 | -0.71* | -0.73* | 0.65 | 0.32 | -0.72 | -0.73* |  |  |
|  | BTS_B | -0.46 | -0.46 | 0.32 | 0.65 | -0.46 | -0.53 | 0.46 | 0.07 | -0.53 | -0.42 | 0.59 |  |
| P-values: ‘***’p< 0.001; ‘**’ p<0.01; ‘*’ p<0.05; ‘.’ p<0.01 | | | | | | | | | | | | | |

**Supplementary Table 4.** Protein sequences of *Oryza*, *Zizania* *latifolia*, and Z. *palustris* sequences for cluster analysis.

| **Gene name** | **Species** | **Gene ID** | **GenBank ID** | **Protein sequence** |
| --- | --- | --- | --- | --- |
| *OsqSH1* | *Oryza sativa* | LOC_Os01g62920.1 | XP_015641948.1 | MSSAAGGGGYGGGQGGGAEHHHHHHGHAGHLLLHHHPQHVAGAAVAAAAAAAGGQMYHVPQHSRREKLRFPPDAGDSPPPHGHGHGHAPQQQQQHGSWPPPPAFYSYASSSSSYSPHSPTLAQAQLVAHGLAPPLPQIPTQNFSLSLSSASSNPPPPQAQPRRQLGGLAQATGPFGPFTGYAAVLGRSRFLGPAEKLFEEICDVGGAASHVDRTISDEGLLDADPMDGVDHDVVDHDLGGADRAAADAGPISGAEQQWKKTKLISMMEEVCKRYRQYYQQVQAVMASFETVAGFSNAAPFAALALRAMAKHFKCLKSMILNQLRNTSNKVAVKDGLNKEIAVFGLAGGSSGGAGLQRANSASAFGQPHNIWRPQRGLPERAVSVLRAWLFEHFLHPYPTDGDKQMLAKQTGLTRNQVSNWFINARVRLWKPMVEEIHNLEMRQMHKHSVVDKGQHSVHHQAQHSSQCSGNPSVPSDSHPGQSSSITRNHNTAASQGFPDELSQMSQSIQGQVSFAYNGLTSQHNIASPHHQHQQVGGVSIGGGNGGVSLTLGLHQNNRVCIAEPLPAALPANLAHRFGLEEVSDAYVMSSFGGQDRHFGKEIGGHLLHDFVG |
| *OsSH1* | *Oryza sativa* | LOC_Os03g44710.1 | NP_001389121.1 | MSAQIVPAPEHVCYVHCNFCNTIFAVSVPSNSMLNIVTVRCGHCTSLLSVNLRGLVQALPAEDHLQDNLKMHNMSFRENYSEYGSSSRYGRVPMMFSKNDTEHMLHVRPPEKRQRVPSAYNRFIKEEIRRIKANNPDISHREAFSTAAKNWAHFPNIHFGLGSHESSKKLDEAIGAPSPQKVQRLY |
| *Ossh4* | *Oryza sativa* | LOC_Os04g57530.1 | XP_015635337.1 | MSGSSADPSPSASTAGAAVSPLALLRAHGHGHGHLTATPPSGATGPAPPPPSPASGSAPRDYRKGNWTLHETLILITANRLDDDRRAGVGGAAAGGGGAGSPPTPRSAEQRWKWVENYCWKNGCLRSQNQCNDKWDNLLRDYKKVRDYESRVAAAAATGGAAAANSAPLPSYWTMERHERKDCNLPTNLAPEVYDALSEVLSRRAARRGGATIAPTPPPPPLALPLPPPPPPSPPKPLVAQQQHHHHGHHHHPPPPQPPPSSLQLPPAVVAPPPASVS |
| *OsSh5* | *Oryza sativa* | LOC_Os05g38120.1 | XP_015637721.1 | MSSAAGGGGGYGGGGGEHQHQQQQHHLLLGQAAGQLYHVPQHSRREKLRFPPDHPAESPPPPPPGSWPLPPAFYSYASSSSSYSPHSPTLAHAQLVAHGMPPGAATSGGAQIPSQNFALSLSSASSNPPPTPRRQFGGGGGGGGAAGPYGPFTGYAAVLGRSRFLGPAQKLLEEICDVGGRPAQLDRGSDEGLLDVDAMDAAGSVDHEMDGSDRAVADAVTVSGAEQQWRKTRLISLMEDVCKRYRQYYQQLQAVVSSFETVAGLSNAAPFASMALRTMSKHFKYLKGIILNQLRNTGKGATKDGLGKEDTTNFGLMGGGAGLLRGNNVNSFSQPHNIWRPQRGLPERAVSVLRAWLFEHFLHPYPTDSDKQMLAKQTGLTRNQVSNWFINARVRLWKPMVEEIHNLEMRQLQKNPSLDKNQLSMQHTQHSSDSSGKPCDPSNSLQGQSSSMTRNHSISASRHIEDGLSQMPHDISGQVSFAYNGLAAHHSIAMAHHHQPDLIGTGGAANAGGVSLTLGLHQNNNRAYIAEPLPAALPLNLAHRFGLEDVSDAYVMSSFGGQDRHFTKEIGGHLLHDFVG* |
| *OsSHAT1* | *Oryza sativa* | LOC_Os04g55560.2 | XP_015636848.1 | MWDLNDSPAAEAAPPPLSPSADDSGASSSSAAAVVEIPDDADDDSAAVVVVTRQFFPPAVPGGGGDPAPGNARAGWLRLAGAAPPVAATGPAASAAVSKKSRRGPRSRSSQYRGVTFYRRTGRWESHIWDCGKQVYLGGFDTAHAAARAYDRAAIKFRGVEADINFSLEDYEDDLKQMSNLTKEEFVHVLRRQSTGFPRGSSKYRGVTLHKCGRWEARMGQFLGKKYVYLGLFDTEEEAARAYDRAAIKCNGKDAVTNFDPSIYAGEFEPPAAATGDAAEHNLDLSLGSSAGSKRGNVDGGGDDEITGGGGGGAGSDQRVPMAFDLDWQTAAARSTKAKFDQNSNHPQMPPVLQVTHLPFSPRHHHQFLSNGDPGTAGGLSLTIGAGMAGHWPPQQQQGWGNAGGMSWPHPPHPPPPPTNAAAAATATAAAASSRFPPYIATQASTWLQKNGFHSLTRPT* |
| *ZlqSH1a* | *Zizania latifolia* | ABZP36_025045 | KAL5220332.1 | MSSAVGGDGYGAGPGDGAEHHHHHHHGHAGHLLLHHHPQHLGGQMYHVPQHSRREKLRFPPDAADSPPPHGHVPQQQQQHGVWPPPPSFYSYASSSSSYSPHSPTLAQAQLVAHGLAPPLPQIPTQNFSLSLSSASSNPPPQPRRPLSGPAQATGPFGPFTGYAAVLGRSRFLGPAEKLFEQICDVGGAAAHVDRAICEDALLDADPMDGADPIDHDVGGADRAAEDPGPISGAEQQWKKTRLISMMEEVCKRYRQYYQQVQAVMASFETVAGFSNAAPFAALALRAMAKHFKYLKGMILNQLRNTCNKPAAKENNKDIAVFGLAGGSGGAGAGAGLQRGNSASAFGQPHNIWRPQRGLPERAVSVLRAWLFEHFLHPYPTDGDKQMLAKQTGLTRNQVSNWFINARVRLWKPMVEEIHNLEMRQMHKHTVLDKGQHGMHHQAQHSSQCSGKPSDPSDSHPRQSSSITRNHNTTASQGFPDELSQMSQSIQGQVNFAYDGLSSQHNIASPHQHQQVTSVGIGSGGAGNGGVSLTLGLHQNNGICMAEPLPTSLPPNLAHRFGLEDVSDAYVMGSFAGPDRHFGKIGGHLLHDFVG |
| *ZlqSH1b* | *Zizania latifolia* | ABZP36_016879 | KAL5228614.1 | MSSAAGGGGGYGAGPAGGAEHQHHGHAGHLLLHHHPHHVASAAAAGGQMYHVPQHSRREKLRFPPDAADSSPPHGHLPQEQQQHGMWPPPPAFYSYASSSSSYSPHSPTLVQAQLMAHGLAPPLPQIPAQNFSLSLSSASSNPPPQPRRQLSGPAQTTGPFGPFTGYAAVLGRSRFLGPTEKLFEEICDVGGAAAHVERTFSDDALLEADPMDGADTVDHEFGGADRAAVHAGPISGAEQQWKKTRLISMMEEVCKRYRQYYQQVQAVMASFETVAGFSNAAPFAALALRAMAKHFKCLKSMILNQLRNTSNKAAAKEGLNNKDVAVFGLAGGSAGAGAVLHRGNNASAFAQPHNIWRPQRGLPERAVSVLRAWLFEHFLHPYPSDGDKQMLAKQTGLTRNQVSNWFINARVRLWKPMVEEIHNLEMRQMHKHSVLDKGQHGMHHHQAQHSSAQHSSQCDGKSSDPSDSHPGQSSSITRNHNNAASQGFPDELSQMSQSIQGQVNFAYNGLSSLHNIASPHQQHQQVAGVGGGGGGVSLTLGLHQNNRVCITEQLPTHALPPNLAHRFGLEDVGDAYVMGSFGGQERHFGKEIGGHFLHDFVG |
| *ZlSH1a* | *Zizania latifolia* | ABZP36_005173 | KAL5209550.1 | MSAQIAPAPEHVCYVHCNFCNTILAVSVPSNSMLNIVTVRCGHCTSLLSVNLRGLVQSLPVEDHFQENLKMHNISFGENYSEYGSSSKYGRVPMMFSKYDPDHMLHVRPPEKRQRVPSAYNRFIKEEIRRIKANNPDISHREAFSTAAKNWAHFPNIHFGLGSHEGSKKLDKAIGAPDPQKVQRLY |
| *ZlSH1b* | *Zizania latifolia* | ABZP36_018673 | KAL5217989.1 | MSAQIAPAAEHVCYVHCNFCNTILAVSVPSNSMLNIVTVRCGHCTSLLSVNLRGLVQSLPVEDHLQENLKMHSISFRENKSEYGSSSKYGRVPMFSKNDPDHMLHVRPPEKRQRVPSAYNRFIKEEIRRIKANNPDISHREAFSTAAKNWAHFPNIHFGLGSNESSKKLDEAELRVLKKFKDSTERGLIKS |
| *Zlsh4a* | *Zizania latifolia* | ABZP36_008339 | KAL5215109.1 | MSASSDPSPSATAASSPLALIRANPHQHPHLTPPSPATGPAPPPASPVSAPRDYRKGNWTLHETLILITAKRLDDDRRAGGGGGGVASAGVAAAGSPPTPRSAEQRWKWVENYCWKNGCLRSQNQCNDKWDNLLRDYKKVRDYESRVAATGAAAASAAAPPHAALPSYWTMERHERKDRNLPTNLAPEVHDALSEVLSRRAARRGGATIAPTSLAPLALPPPPPPPPSPPKPLIAQQQHHHHHHPPPPLPPVIAPPPASVSAEEMSGSSSSEEDNGSGGEPEAKRRRLNRLGSSVVRSATVVARTLVACEEKRERRHRELLQLEERRLRLEEERTEVRRQGFAGLVSAVNSLSSAIHALVSDHRSGDSSR |
| *Zlsh4b* | *Zizania latifolia* | ABZP36_004261 | KAL5216938.1 | MSASSDPSASSSSAAASPLALIRAHTHHPHPHFTPPSPSTGPAPPPPSPVSAPRDYRKGNWTLHETLILITAKRLDDDRRAGGGSGAGAGAGAGAAAAGSPPPTPRSAEQRWKWVENYCWKNGCLRSQNQCNDKWDNLLRDYKKVRDYESRVAATAGAAAAGASTGAAPLATLLPSYWTMERHERKDRNLPTNLAPEVYDALSDVLSRRAARRGGVTIAPTPPPPLALPPPPPPPPPPSPPKPLITQHQHHPPHHPPLPPAVAPPPASVSAEEEMSGSSESGEEEGSGGEPESKRRRLSRLGSSVVRSATVVARTLVACEEKRERRHRELLQLEERRLRLEEERTEVRRQGFAGLISAINSLSGAIHALVSDHRSGDSSR |
| *ZlSh5a* | *Zizania latifolia* | ABZP36_013876 | KAL5202924.1 | MSSSAGGGVGGYGGGAEQHQQLLLGQGAGQLYHVPQHSRREKLRFPPDPTDSSPLPGSWPLPPPFYSYASSSSSYSPHSPTLAHAQVQLVAHGMPGTASGGAQIPSQNFALSLSSASSNPPPTPRRQFGGSGGAAGPYGPFTGYAAVLGRSRFLGPAQKLLEEICDVGGRSAQLDRGSDEGLLHVDAMDAAGSVDYEMDDSDRAAAEAVTVSGAEQQWRKTRLISLMEDVCKRYKQYYQQLQAVISSFETVAGLSNAAPFASVALRTMSKHFKYLKGMILNQLRNTSKGAAKDGLGKEDVANFGLMGGGASLLGGNNVNSFSQPHNIWRPQRGLPERAVSVLRAWLFEHFLHPYPTDSDKQMLAKQTGLSRNQVSNWFINARVRLWKPMVEEIHNLEMKQLQKNPSLDKNQLSMQHTQHSSDSSGKPSDPSNSLQGQGSSMTRNHSVNASRHIEDGLSQMPHDISGQVSFAYNGLTSHHNNALSHPQQQPDLIGAGGAANAAGVSLTLGLHQNNRAYIAEPLPAALPLNLAHRFGLEDVSDAYVMGSFGGQDRHFTKEIGGHLLHDFVG |
| *ZlSh5b* | *Zizania latifolia* | ABZP36_030161 | KAL5231385.1 | MSSAAGGGGYGGGAEQHQHQQLLLGQAAGQLYHVPQHSRREKLRFPPDPADSSPPLGSWPPPPPFYSYASSSSSYSPHSHAQAQLVAHGIPGTASGGAQIPNQNFALSLSSASSNPPPTPRSQFGGSGGAAGPYGPFTGYAAVLGRSRFLGPAQKLLEEICDVGGRPSQLNRGSDEGLLDLDAMDATGSVDHDMDAGDRAATEAVTVSGAEQQWRKTRLISLMEEVCKRYKQYYQQLQAVISSFETVAGLSNAAPFASMALRTMSKHFKYLKGMILNQLRNTSKDAAKDGLGKEDVPDFGLMGGGASLLRGNNVNSFSQPHNIWRPQRGLPERAVSVLRAWLFEHFLHPYPTDSDKQMLAKQTGLTRNQVSNWFINARVRLWKPMVEEIHSLEMRQLQKNPSLDKNQLSMQHTQHLSDSSGKPSDPSNSQLQGQSSSMARNHGVSAASRHIDDGLAQMPHDISGQVSFAYNGLTSHHTIAVSHPQQPELIGTGSAANSGGVSLTLGLHQNNRAYIAEPLPAALPLNLAHRFGLEDVSDAYVMGSFGSQDRHFTKEMGGHLLHDFVG |
| *ZlSHAT1a* | *Zizania latifolia* | ABZP36_003055 | KAL5213903.1 | MWDLNDSPAAEGAPPPQLSPSADDSGASSSSAAAVVEIPDDADDDSATAAGAVVTRQFFPPAPPGEPAPGNVRAGWLRLAGTAPPAAAAGGAAAAAAAAASKKSRRGPRSRSSQYRGVTFYRRTGRWESHIWDCGKQVYLGGFDTAHAAARAYDRAAIKFRGVEADINFSLEDYEDDLKQMSNLSKEEFVHVLRRQSTGFPRGSSKYRGVTLHKCGRWEARMGQFLGKKYVYLGLFDTEEEAARAYDRAAIKCSGKDAMTNFDQSIYAGELEPAAVATGDGEHNLDLSLGSSAGSKRGSLHGGGDDESAGGSAAGSDQRVAMAFDLDWQTAARSTKAKFDQNSKHPQMPPVLQVSHLPFSPRHHQFVSNGDPGTAGGLPLTIGGVGGAGGGHWPPQPQLHGWGSGGGMNWPMPPHPPPPANDAAATATATAAAASSRFPPYIATQASSWLQKNGYHSLTRPT |
| *ZlSHAT1b* | *Zizania latifolia* | ABZP36_007933 | KAL5216532.1 | MWNLNDSPAAEGQPPPPALSPSGDDSGASSSSAAAVVEIPDDADDDSVAAADADADAVVTRQFFPPAAPGEPAPGNTRAGWLRLARAAPPAPAAGVAAAAAAAASKKSRRGPRSRSSQYRGVTFYRRTGRWESHIWDCGKQVYLGGFDTAHAAARAYDRAAIKFRGVEADINFSLEDYEDDLKQMSNLTKEEFVHVLRRQSTGFPRGSSKYRGVTLHKCGRWEARMGQFLGKKYVYLGLFDTEEEAARAYDRAAIKCNGKDAVTNFDPSIYAGEFDPMAAATGDGEHNLDLSLGSSAGSKRGSLDGGGDDESAGGAAAGSDERVHMAFDLDWQTAARSTKAKFDQNLKHPQMPPVLQVSHLPFSPRHHQVVREQWRSGDSGRPVSDDRRRGRRGRRALASSADASAATARLGQRRRRHELADAAAPAANQRRRRRRRNRYSSCSIITIPTLHCNASLKLAAEEWVPFPDPTHLDDDHQPCASVVSPWKRPTIRSIERRCVSLTQGNYSIATAT |
| *ZpqSH1a* | *Zizania palustris* | ZPchr0001g31318 | KAG8054755 | MSSAAGAGGYGGAGGAEHQRHGHAGHLLLQHHPHHVAGAAAAGGQMYHVPQHSRREKLRFPPDAADASPPHGHVPVPQEEQPQHGMWPPPPAFYSYASSSSSYSPHSPTLVQAQLMAHGLAPPLPQIPTHNFSLTLSSASSNPPPQPRRQLSGPAQATGPFGPFTGYAAVLGRSRFLGPTEKLFEEICDVGGAAAHVERTFSDDALLEADPMDGADTVDHEFADRAAADAGPISGAEQQWKKTRLISMMEEVCKRYRQYYQQVQAVMASFETVAGFSNAAPFAALALRAMAKHFKCLKNMILNQLRNTSNKAAAKDGLSNKDVAVFGLAGGSVGAGAGLQRGNNASAFVQPHNIWRPQRGLPERAVSVLRAWLFEHFLHPYPSDGDKQMLAKQTGLTRNQVSNWFINARVRLWKPMVEEIHNLEMRQMHKHSVLDKGQHGTHHQAQHSSQCGGKPSDPSDSHPGQSSSITRNHNNAASHGFPDELSQMSQSIQEQAAVNFAYNGFSSLHNVASPHQQHQQQVAGGGNNGNGGVSLTLGLHQNNRVCITEPLPTHALPPNLAHRFGLEDVSDAYVMGSFGGQERHFGKEIGGHFLHDFVG |
| *ZpqSH1b* | *Zizania palustris* | ZPchr0007g5872 | KAG8078479 | MSSAVGGDGYGVGPGDGAEQHGHVHGHAGHLLLHHHPQHLPAVAAAGGQMYHVPQHSRREKLRFPPDPADSPPLHGHVPQQQQHGVWPPPPSFYSYASSSSSYSPHSPTLAQAQLVAHGLAPPLPQIPTQNFSLSLSSASSNPPTQQRRPPSGPAQATGPFGPFTGYAAVLGRSRFLGPAEKLFEQICDVGGAAAHVHHTICEDALLDADPMDGADPVDHDLGSADRAAEDPGPISGAEQQWKKTRLISMMEEVCKRYRQYYQQVQAVMASFETVAGFSNAAPFTALALRAMAKHFKYLKGMILNQLRNTSSKQPGAKESNKDVAMFGLAGGSAGLQRGNSASAFSQPPNIWRPQRGLPERAVSVLRAWLFEHFLHPYPTDGDKQMLAKQTGLTRNQVSNWFINARVRLWKPMVEEIHNLEMRQLHKHSVLDKGQHGMHQQAQHSSQCSGKPSDPSDSHPGQSSSITRNHNATASHGFPDELSQMSQSIQGQVNFAYDGLSSQHNIASPHQHRQVAAIGIGSGVAGAGTGNGGVSLTLGLHQNNRICMAEPLPNALPPNLAHRFGLEDVSDAYVMGSFAGPDRHFGKIGGHLLHDFVG |
| *ZpSH1* | *Zizania palustris* | ZPchr0003g18426 | KAG8062892 | MSAQIAPAAEHVCYVHCNFCNTILAVSVPSNSMLNIVTVRCGHCTSLLSVNLRGLLQSLPVEDHLQENLKMHSISFRENNSEYGSSSKYGRVPMFSKNDPDHMLHVRPPEKRQRVPSAYNRFIKEEIRRIKANNPDISHREAFSTAAKNWAHFPNIHFGLGSNESSKKLDEAIGAAPGSKKVQRLY |
| *Zpsh4a* | *Zizania palustris* | ZPchr0004g38781 | KAG8066642 | MSASSDPSPSSSSAAASPLALIRAHTHHPHPHVTPLSPSTGPAPPPPSPVSAPRDYRKGNWTLHETLVLITAKRLDDDRRAGGAGAGAGAAAAGSPPPTPRSAEQRWKWVENYCWKNGCLRSQNQCNDKWDNLLRDYKKVRDYESRVAATAGAAASGASTGAATLAAVLLPSYWTIERHDRKDRNLPTNLAPEVYDALSEVLSRRAARRGGVTIAPTPPPPLALPPPPPPPPPSPPKPLITQQQQQQQHHPPLPPAVAPPPASVSAEEEEMSGEQEEGEEEEEEGSGGEPESKRRRLGSSVVRSATVVARTLVACEEKRERRHRELLQLEERRLRLEEERTEVRRQGFAGLISAINNLSGAIHALVSDHRSGDSSR |
| *Zpsh4b* | *Zizania palustris* | ZPchr0458g22499 | KAG8043886 | MSASSDPSASAAAASSPLALIRANPHLTPPSPVPATGPAPPPPSPVSAPRDYRKGNWTLHETLILITAKRLDDDRRAGGGGGGGGVAGAGVAAVGSPPTPRSAEQRWKWVENYCWKNGCLRSQNQCNDKWDNLLRDYKKVRDYESPEEEMSVSSESEEEDGSGGEPEAKRRRLNRLGSSVVRSATVVARTLVACEEKRERRHRELLQLEERRLRLEEERTEVRRQGFAGLVSAVNSLSSAIHALVSDHRSGDSSR |
| *Zpsh4c* | *Zizania palustris* | ZPchr0458g22823 | KAG8043873 | MSASSDPSASAAAASSPLALIRANPHLTPPSPVPATGPAPPPPSPVSAPRDYRKGNWTLHETLILITAKRLDDDRRAGGGGGGGGVAGAGVAAVGSPPTPRSAEQRWKWVENYCWKNGCLRSQNQCNDKWDNLLRDYKKVRDYESRIAAAAASAAAAPPHAALPSYWTLERHERKDRNLPTNLAPEVHDALSEVDVYMLCGFVAEEEMSVSSESEEEDGSGGEPEAKRRRLNRLGSSVVRSATVVARTLVACEEKRERRHRELLQLEERRLRLEEERTEVRRQGFAGLVSAVNSLSSAIHALVSDHRSGDSSSDSPLRALGELCEKARSIIASHAAKRKQRRESSSSSGSQQGALESRLRPTHYAPRRGVPTSAAGGCPASPELQ |
| *ZpSh5a* | *Zizania palustris* | ZPchr0001g31104 | KAG8052160 | MDSFPYPCPHGHMRESKGRAWVRELWRRHAFGRAMTLHLHTTTPRRATPQKRPGGAQSKDIHQIRSARAAGQSYRSIDLGNRRQLAPSAEPPRCSTTTTTALFSLRILSPGRLRRTACVRACAHRSERAACPPRREEDWEDTEAARPSSTSTSNCCLGRVRPAGQLYHVPQHSRREKLRFPPDPTDSSPPPGSWPPPPPFYSYASSSSSYSPHSPTLAHAPVQLVAHGMPGTASGGAQIPSQNFALSLSSASSNLFLGPAQKLLEEICDVGGRSAQLDRGSGEGLLPVDAMDAAGSVDHEMDDSDRAAAEAVTVCGAEQQWRKTRLISLMEDNCLHLKL |
| *ZpSh5b* | *Zizania palustris* | ZPchr0005g15825 | KAG8068426 | MSSAPGGGGYGGGADQNQHQQLLLGQAAGQLYHVPQHSRREKLRFPPDHADSSPPPGSWPPPPPFYSYASSSSSYSPHSHAQAQLVAQGMSGTASGGAQIPSQNFALSLSSASSNPPPTPRRQFGGSGGAAGPYGPFTGYAAVLGRSRFLGPAQKLLEEICDVGGRPSQLNRGSDEGLLDLDAMDATGSVDHDMDASDHAAAEAVTVSGAEQQWRKTRLISLMEEVCKRYKQYYQQLQAVISSFETVAGLSNAAPFASMALRTMSKHFKYLKGMILNQLRNTSKGATKDCLGKEDVPDFGLMGGGASLLRGNNVNSFSQPHNIWRPQRGLPERAVSVLRAWLFEHFLHPYPTDSDKQMLAKQTGLTRNQVSNWFINARVRLWKPMVEEIHSLEMRQLQKNPSLDKNQLSMQHTQHLSDSSGKLSDASNSQLQGQSSSMARNHGINAVRHIDDGLSQMPHDISGQVSFAYNGITSHHTIAVSHPQQPDLIGTGSAANAGGVSLTLGLHQNNRAYIAEPLPAALPLNLAHRFGLEDVSDGYVMGSFGSQDRHFPKEMGGHLLHDFVG |
| *ZpSh5c* | *Zizania palustris* | ZPchr0010g10516 | KAG8085781 | MSSSAGGGLGGYGGGAAEQHQHQQLLLGQGAGQLYHVPQHSRREKLRFPPDPTDSSLPPGSWPPPPPFYSYASSSSSYSPHSPTLAHAPAQLVAHGMPGTASGEAQIPSQNFALSLSSASSNPPPTPRTQFGGSGGAAGPYGPFTGYAAVLGRSRFLGPAQKLLEEICDVGGRSAQLDRGSDEGLLPVDAMDAAGSVDHEMDDSDRAAAEAVTVCGAEQQWRKTRLISLMEDVCKRYKQYYQQLQAVISSFETVAGLSNAAPFASIALRTMSKHFKYLKGMILNQLRNTSKGAAKDGLGKEDVANFGLMGGGASLLRGNNVNSFSQPHNIWRPQRGLPERAVSVLRAWLFEHFLHPYPTDSDKQMLAKQTGLSRNQVSNWFINARVRLWKPMVEEIHNLEMKQLQKNPSLDKNQLPMQHTQHSSDSSGKPSDPSNSLQGQGSSMTRNHSVNATRHIEDGLSQMPHDISGQVSFAYNGLTSHHSIALSHPQQQHDLIGAGGGAANAAGVSLTLGLHQNNRAYIAEPLPAALPLNLAHRFGLEDVSDPYVMGSFGGQDRHFAKEIGGHLLHDFVGLQSTELQGKGVRVDMNRGTELKLRGTARLDIVAGSTEFVTESFNRIKPFLKDFMERGVLVVPFSMDGNTLSLQFDIGAGTVAIGPQVADPVSQSSTRPELEMN |
| *ZpSh5d* | *Zizania palustris* | ZPchr0010g7757 | KAG8085804 | MSSSAGGGLGGYGGGAAEQHQHQQLLLGQGAGQLYHVPQHSRREKLRFPPDPTDSSLPPGSWPPPPPFYSYASSSSSYSPHSPTLAHAPAQLVAHGMPGTASGEAQIPSQNFALSLSSASSNPPPTPRTQFGGSGGAAGPYGPFTGYAAVLGRSRFLGPAQKLLEEICDVGGRSAQLDRGSDEGLLPVDAMDAAGSVDHEMDDSDRAAAEAVTVCGAEQQWRKTRLISLMEDVCKRYKQYYQQLQAVISSFETVAGLSNAAPFASIALRTMSKHFKYLKGMILNQLRNTSKGAAKDGLGKEDVANFGLMGGGASLLRGNNVNSFSQPHNIWRPQRGLPERAVSVLRAWLFEHFLHPYPTDSDKQMLAKQTGLSRNQVSNWFINARVRLWKPMVEEIHNLEMKQLQKNPSLDKNQLPMQHTQHSSDSSGKPSDPSNSLQGQGSSMTRNHSVNATRHIEDGLSQMPHDISGQVSFAYNGLTSHHSIALSHPQQQHDLIGAGGGAANAAGVSLTLGLHQNNRAYIAEPLPAALPLNLAHRFGLEDVSDPYVMGSFGGQDRHFAKEIGGHLLHDFVGGCRHHFFFLLQSSAGRRQKAVAQRCRVLNCSLQGKGVRVDMNRGTELKLRGTARLDIVAGSTEFVTESFNRIKPFLKDFMERGVLVVPFSMDGNTLSLQFDIGAGTVAIGPQVADPVSQSSTRPELEMN |
| *ZpSHAT1a* | *Zizania palustris* | ZPchr0004g38673 | KAG8066465 | MPRHSSTRRGGGEEVRQEEEDELVPRLVFFDRWPLEIGSEAVPHRFFSPRFYPTLRLDRSIPFFQSLAQQLQRPGRPPSHAFGVLCCAPRLGEAFLRRRDRQKRKYLHSPTRQQLLPFLFCSLFLSLSRSRSPLALAFLSIYLLRCQLPRPNWGNRGPAMWDLNDSPAAERAPTQLSPSADDSGASSSSAAAVVEIPDDGDDDSAAAADAVVTRQFFPPAAPGEPAPGNARAGWLRLAGAAPPAAAAGAAAAAAAASSKKSRRGPRSRSSQYRGVTFYRRTGRWESHIWDCGKQVYLGGFDTAHAAARAYDRAAIKFRGVEADINFSLEDYEVDLKQMSNLSKEEFVHVLRRQSTGFPRGSSKYRGVTLHKCGRWEARMGQFLGKKYVYLGLFDTEEEAARAYDRAAIKCNGKDAMTNFDPSIYAGELEPAAAATGDGEHNLDLSLGSSTGSKRGSLDGGGDDESAGASATGLDQRVPMAFDLDWQTVARSTKAKFDQNSKHPQMPPVLQVSHLPFSPRHHQFVSTGDPGTAGGPSLTIGGVGGAGGGHWPPHQPPQLHDWGSGAAGMNWPMPPHPPSPAAAATATAAAASSRFPPYIATQASSWLQKNGFHSLTRPT |
| *ZpSHAT1b* | *Zizania palustris* | ZPchr0458g22566 | KAG8043734 | MAPPGRGRAAGPCAGVAAAASASASKKSRRGPRSRSSQYRGVTFYRRTGRWESHIWDCGKQVYLGGFDTAHAAARAYDRAAIKFRGVEADINFSLEDYEDDLKQMSNLTKEEFVHVLRRQSTGFPRGSSKYRGVTLHKCGRWEARMGQFLGKKYVYLGLFDTEEEAARAYDRAAIKCNGKDAVTNFDPSIYAGEGGEFDPMAAATGDAEHNLDLSLGSSAGSKRGNLDGGGDDESAGGAVAGSDERVPMAFDLDWQTAARSTKAKFDQNSKHPQMPPVLQVSHLPFSPRHHQFVSNGDPGTAGGLSLTIGGVGGAGGGHWSPQPPPQMQLHGWGSGGGGMNWPMPPHLPPPPSTNAATAATATAAAASSRFPPYIATQASSWLQKNGFHSLTRPT |

**Supplementary Table 5.** Identification of orthogroups between *Oryza sativa*, *Zizania palustris*, and *Z.latifolia* .

| **Name** | **Orthogroup** | ***O. sativa* GenBank ID** | ***Z. latifolia GenBank ID*** | **Description and domain** |
| --- | --- | --- | --- | --- |
| *ZpSH1* | OG0010120 | NP_001389121.1 | KAL5209550.1, KAL5217989.1 | SH1 homolog; YABBY Transcription factor |
| *ZpSh5a* | NA | NA | NA | No orthogroup; unknown domain |
| *ZpSh5b* | OG0013611 | XP_015637721.1 | KAL5202924.1, KAL5231385.1 | Sh5 candidate; BEL1-like homeobox domain |
| *ZpSh5c* | OG0026538 | NA | NA | No orthogroup, possible recent duplication |
| *ZpSh5d* | OG0026538 | NA | NA | No orthogroup, possible recent duplication |
| *ZpqSH1a* | NA | NA | NA | No orthogroup, incomplete or low sequence similarity |
| *ZpqSH1b* | OG0008858 | XP_015641948.1 | KAL5220332.1, KAL5228614.1 | qSH1 homolog; BEL1-like homeobox domain |
| *ZpSHAT1a* | NA | NA | NA | No orthogroup; AP2 transcription factor but weak support |
| *ZpSHAT1b* | OG0003844 | NP_001411211.1, XP_015636848.1 | KAL5213903.1, KAL5216532.1 | SHAT1 homolog; AP2 transcription factor |
| *Zpsh4a* | OG0004851 | XP_015635337.1 | KAL5215109.1, KAL5216938.1 | sh4 homolog, likely recent duplicates; trihelix transcription factor |
| *Zpsh4b* | OG0004851 | XP_015635337.1 | KAL5215109.1, KAL5216938.1 | sh4 homolog, likely recent duplicates; trihelix transcription factor |
| *Zpsh4c* | OG0004851 | XP_015635337.1 | KAL5215109.1, KAL5216938.1 | sh4 homolog, likely recent duplicates; trihelix transcription factor |

Legend: Candidate shattering homologs for Northern Wild rice (*Zizania palustris*) with a percent of protein identity ≥ 70% were assigned to orthogroups using OrthoFinder version 2.5.4 (Emms and Kelly, 2015).

**Supplementary Table 6.** Pfam domain annotations of putative *Zizania palustris* homologs of *Oryza sativa* seed shattering-related genes as identified by InterProScan.

| **Name** | **GenBankID** | **Amino acid #** | **Pfam** | **Protein domain** | **Start (bp)** | **Finish (bp)** | **E-value** | **IPR** | **Protein domain** | **GO term** |
| --- | --- | --- | --- | --- | --- | --- | --- | --- | --- | --- |
| OsqSH1 | XP_015641948.1 | 612 | PF05920 | Homeobox KN domain | 388 | 427 | 1.10E-16 | IPR008422 | KN homeodomain | GO:0003677(InterPro)\|GO:0006355(InterPro) |
| OsSh5 | XP_015637721.1 | 580 | PF05920 | Homeobox KN domain | 356 | 395 | 6.10E-17 | IPR008422 | KN homeodomain | GO:0003677(InterPro)\|GO:0006355(InterPro) |
| ZlqSH1a | KAL5220332.1 | 595 | PF05920 | Homeobox KN domain | 370 | 409 | 1.00E-16 | IPR008422 | KN homeodomain | GO:0003677(InterPro)\|GO:0006355(InterPro) |
| ZlqSH1b | KAL5228614.1 | 603 | PF05920 | Homeobox KN domain | 374 | 413 | 9.50E-17 | IPR008422 | KN homeodomain | GO:0003677(InterPro)\|GO:0006355(InterPro) |
| ZpqSH1a | KAG8054755.1 | 599 | PF05920 | Homeobox KN domain | 372 | 411 | 9.40E-17 | IPR008422 | KN homeodomain | GO:0003677(InterPro)\|GO:0006355(InterPro) |
| ZpqSH1b | KAG8078479.1 | 598 | PF05920 | Homeobox KN domain | 369 | 408 | 1.00E-16 | IPR008422 | KN homeodomain | GO:0003677(InterPro)\|GO:0006355(InterPro) |
| ZpSh5b | KAG8068426.1 | 565 | PF05920 | Homeobox KN domain | 341 | 380 | 5.90E-17 | IPR008422 | KN homeodomain | GO:0003677(InterPro)\|GO:0006355(InterPro) |
| ZpSh5c | KAG8085781.1 | 676 | PF05920 | Homeobox KN domain | 348 | 387 | 8.00E-17 | IPR008422 | KN homeodomain | GO:0003677(InterPro)\|GO:0006355(InterPro) |
| ZpSh5d | KAG8085804.1 | 702 | PF05920 | Homeobox KN domain | 348 | 387 | 8.40E-17 | IPR008422 | KN homeodomain | GO:0003677(InterPro)\|GO:0006355(InterPro) |
| OsqSH1 | XP_015641948.1 | 612 | PF07526 | Associated with HOX | 184 | 319 | 1.40E-32 | IPR006563 | POX domain | - |
| OsSh5 | XP_015637721.1 | 580 | PF07526 | Associated with HOX | 158 | 290 | 3.80E-32 | IPR006563 | POX domain | - |
| ZlqSH1a | KAL5220332.1 | 595 | PF07526 | Associated with HOX | 166 | 299 | 2.00E-29 | IPR006563 | POX domain | - |
| ZlqSH1b | KAL5228614.1 | 603 | PF07526 | Associated with HOX | 170 | 303 | 8.50E-33 | IPR006563 | POX domain | - |
| ZpqSH1a | KAG8054755.1 | 599 | PF07526 | Associated with HOX | 170 | 301 | 1.20E-32 | IPR006563 | POX domain | - |
| ZpqSH1b | KAG8078479.1 | 598 | PF07526 | Associated with HOX | 170 | 303 | 2.00E-30 | IPR006563 | POX domain | - |
| ZpSh5b | KAG8068426.1 | 565 | PF07526 | Associated with HOX | 143 | 275 | 1.10E-33 | IPR006563 | POX domain | - |
| ZpSh5c | KAG8085781.1 | 676 | PF07526 | Associated with HOX | 150 | 282 | 4.70E-32 | IPR006563 | POX domain | - |
| ZpSh5d | KAG8085804.1 | 702 | PF07526 | Associated with HOX | 150 | 282 | 5.00E-32 | IPR006563 | POX domain | - |

**Supplementary Table 7.** Tukey's Honestly Significant Difference test for Northern Wild Rice (NWR; *Zizania palustris*) relative gene expression (log_2_ transformed) of candidate genes and populations.

| **ANOVA by gene** | **Timepoint or genotype** | **Adjusted means** |
| --- | --- | --- |
| *ZpSh5a* | 28 DAA | 12.01 a |
|  | 35 DAA | 3.52 ab |
|  | 18 DAA | 2.68 b |
|  | 01 DAA | 0.51 b |
|  | 07 DAA | 0.27 b |
| *ZpSh5c* | Wild type | 22.53 a |
|  | FY-C20 | 14.77 a |
|  | Itasca-C12 | 8.83 a |
| **ANOVA by genotypes** | **Gene** | **Adjusted means** |
| Wild type | *ZpSh1* | 24.02 a |
|  | *ZpSh5a* | 2.04 b |
|  | *ZpSh5b* | 0.96 b |
|  | *ZpSh5c* | 22.53 a |
| Itasca-C12 | *ZpSh1* | 21.58 a |
|  | *ZpSh5a* | 1.40 b |
|  | *ZpSh5b* | 0.31 b |
|  | *ZpSh5c* | 8.83 ab |
| Legend: DAA **=** days after anthesis. Tukey’s HSD test = means with the same letter are not statistically significantly different at a threshold of P = 0.05. | | |

**Supplementary Table 8.** Pearson correlation coefficients and corresponding p-values for days after anthesis for female and male tissues of Northern Wild Rice (NWR; *Zizania palustris*) across 5 genes and 2 cultivated and one wild type populations

| Tissue / DAA | Male / 7 | Female / 1 | Female / 7 | Female / 18 | Female / 28 | Female / 35 |
| --- | --- | --- | --- | --- | --- | --- |
| Male / 7 |  |  |  |  |  |  |
| Female / 1 | 0.82* |  |  |  |  |  |
| Female / 7 | 0.80* | 0.78* |  |  |  |  |
| Female / 18 | 0.38* | 0.2 | 0.39* |  |  |  |
| Female / 28 | 0.34 | 0.13 | 0.35 | 0.25 |  |  |
| Female / 35 | 0.06 | -0.05 | -0.02 | 0.58* | 0.04 |  |

**Supplementary Figure 1.** Days from planting to anthesis for three Northern Wild Rice (*Zizania palustris*) populations grown in a greenhouse in St. Paul, Minnesota.

**Supplementary Figure 2.** Protein sequence alignment and a neighbor-joining tree with 100 bootstraps of *Oryza sativa, Zizania palustris* and *Z. latifolia* homologs of *SH1* = *Shattering1* (Lin et al., 2012) using MAFFT v7.511 with the E-INS-i algorithm (Katoh and Standley, 2013).

**Supplementary Figure 3.** Protein sequence alignment and a neighbor-joining tree with 100 bootstraps of *Oryza sativa, Zizania palustris* and *Z. latifolia* homologs of *qSH1* = QTL of seed shattering in chromosome 1 (Konishi et al., 2006) using MAFFT v7.511 with the E-INS-i algorithm (Katoh and Standley, 2013).

**Supplementary Figure 4.** Protein sequence alignment and a neighbor-joining tree with 100 bootstraps of *Oryza sativa, Zizania palustris* and *Z. latifolia* homologs of *SHAT1* = *Shattering Abortion1* (Zhou et al, 2012) using MAFFT v7.511 with the E-INS-i algorithm (Katoh and Standley, 2013).

**Supplementary Figure 5.** Protein sequence alignment and a neighbor-joining tree with 100 bootstraps of *Oryza sativa, Zizania palustris* and *Z. latifolia* homologs of *sh4* (Li et al, 2006) using MAFFT v7.511 with the E-INS-i algorithm (Katoh and Standley, 2013).

**Supplementary Figure 6.** Protein sequence alignment and a neighbor-joining tree with 100 bootstraps of *Oryza sativa, Zizania palustris* and *Z. latifolia* homologs of *Sh5* (Yoon et al, 2014) using MAFFT v7.511 with the E-INS-i algorithm (Katoh and Standley, 2013).
